# LIM and cysteine-rich domains 1 (LMCD1) regulates skeletal muscle hypertrophy, calcium handling, and force

**DOI:** 10.1186/s13395-019-0214-1

**Published:** 2019-10-31

**Authors:** Duarte M. S. Ferreira, Arthur J. Cheng, Leandro Z. Agudelo, Igor Cervenka, Thomas Chaillou, Jorge C. Correia, Margareta Porsmyr-Palmertz, Manizheh Izadi, Alicia Hansson, Vicente Martínez-Redondo, Paula Valente-Silva, Amanda T. Pettersson-Klein, Jennifer L. Estall, Matthew M. Robinson, K. Sreekumaran Nair, Johanna T. Lanner, Jorge L. Ruas

**Affiliations:** 1 Molecular & Cellular Exercise Physiology, Department of Physiology and Pharmacology, Karolinska Institutet, Biomedicum, SE-171 77 Stockholm, Sweden; 2 Molecular Muscle Physiology and Pathophysiology, Department of Physiology and Pharmacology, Karolinska Institutet, Biomedicum, SE-171 77 Stockholm, Sweden; 3 Present Address: Faculty of Health, York University, School of Kinesiology and Health Science, Toronto, Ontario Canada; 4 Present Address: Computer Science and Artificial Intelligence Laboratory, Massachusetts Institute of Technology, Cambridge, MA 02139 USA; 5 School of Health Sciences, Örebro University, Örebro, Sweden; 6 Present Address: Karp Research Building, Boston, MA 02115 USA; 7 Division of Cardiovascular and Metabolic Disease, Institut de recherches cliniques de Montreal (IRCM), Montreal, QC Canada; 8 Division of Endocrinology, Diabetes and Nutrition, Mayo Clinic, Rochester, MN 55905 USA

**Keywords:** Calcineurin, Calcium, Force, Hypertrophy, LMCD1, Skeletal muscle

## Abstract

**Background:**

Skeletal muscle mass and strength are crucial determinants of health. Muscle mass loss is associated with weakness, fatigue, and insulin resistance. In fact, it is predicted that controlling muscle atrophy can reduce morbidity and mortality associated with diseases such as cancer cachexia and sarcopenia.

**Methods:**

We analyzed gene expression data from muscle of mice or human patients with diverse muscle pathologies and identified LMCD1 as a gene strongly associated with skeletal muscle function. We transiently expressed or silenced LMCD1 in mouse *gastrocnemius* muscle or in mouse primary muscle cells and determined muscle/cell size, targeted gene expression, kinase activity with kinase arrays, protein immunoblotting, and protein synthesis levels. To evaluate force, calcium handling, and fatigue, we transduced the *flexor digitorum brevis* muscle with a LMCD1-expressing adenovirus and measured specific force and sarcoplasmic reticulum Ca^2+^ release in individual fibers. Finally, to explore the relationship between LMCD1 and calcineurin, we ectopically expressed *Lmcd1* in the *gastrocnemius* muscle and treated those mice with cyclosporine A (calcineurin inhibitor). In addition, we used a luciferase reporter construct containing the *myoregulin* gene promoter to confirm the role of a LMCD1-calcineurin-myoregulin axis in skeletal muscle mass control and calcium handling.

**Results:**

Here, we identify LIM and cysteine-rich domains 1 (LMCD1) as a positive regulator of muscle mass, that increases muscle protein synthesis and fiber size. LMCD1 expression in vivo was sufficient to increase specific force with lower requirement for calcium handling and to reduce muscle fatigue. Conversely, silencing LMCD1 expression impairs calcium handling and force, and induces muscle fatigue without overt atrophy. The actions of LMCD1 were dependent on calcineurin, as its inhibition using cyclosporine A reverted the observed hypertrophic phenotype. Finally, we determined that LMCD1 represses the expression of myoregulin, a known negative regulator of muscle performance. Interestingly, we observed that skeletal muscle LMCD1 expression is reduced in patients with skeletal muscle disease.

**Conclusions:**

Our gain- and loss-of-function studies show that LMCD1 controls protein synthesis, muscle fiber size, specific force, Ca^2+^ handling, and fatigue resistance. This work uncovers a novel role for LMCD1 in the regulation of skeletal muscle mass and function with potential therapeutic implications.

## Background

Skeletal muscle is one of the most dynamic tissues in the human body comprising approximately 40% of the total body weight and 50–70% of total body protein content. Skeletal muscle mass is strictly dependent on the proper balance between protein degradation and synthesis [1] and its loss is associated with weakness and fatigue. Muscle wasting can be associated with a primary disease (e.g., cancer cachexia), or happen as a consequence of prolonged disuse, or aging (sarcopenia). Conversely, muscle hypertrophy is characterized by an increase in protein synthesis to support increased skeletal muscle mass. However, this increase in muscle size does not always correspond to increased specific force [2]. For example, two different mouse models with mutations in the myostatin gene, a known regulator of muscle growth, show robust increases in skeletal muscle mass but with lower specific force than wild-type animals [3].

Although our understanding of the mechanisms that regulate muscle mass maintenance is still largely incomplete, several key players have been identified. For example, muscle-secreted insulin-like growth factor 1 (IGF-1) can increase protein synthesis and ribosome biogenesis through the activation of the phosphatidylinositol 3-kinase/AKT pathways [4]. Conversely, myostatin acts as a negative regulator of muscle mass by promoting protein degradation [5]. Interestingly, peroxisome proliferator-activated receptor gamma coactivator-1alpha4 (PGC-1α4), which is stimulated by resistance exercise training, can induce muscle hypertrophy by simultaneously regulating the IGF-1 and myostatin pathways [6].

**Fig. 1:**
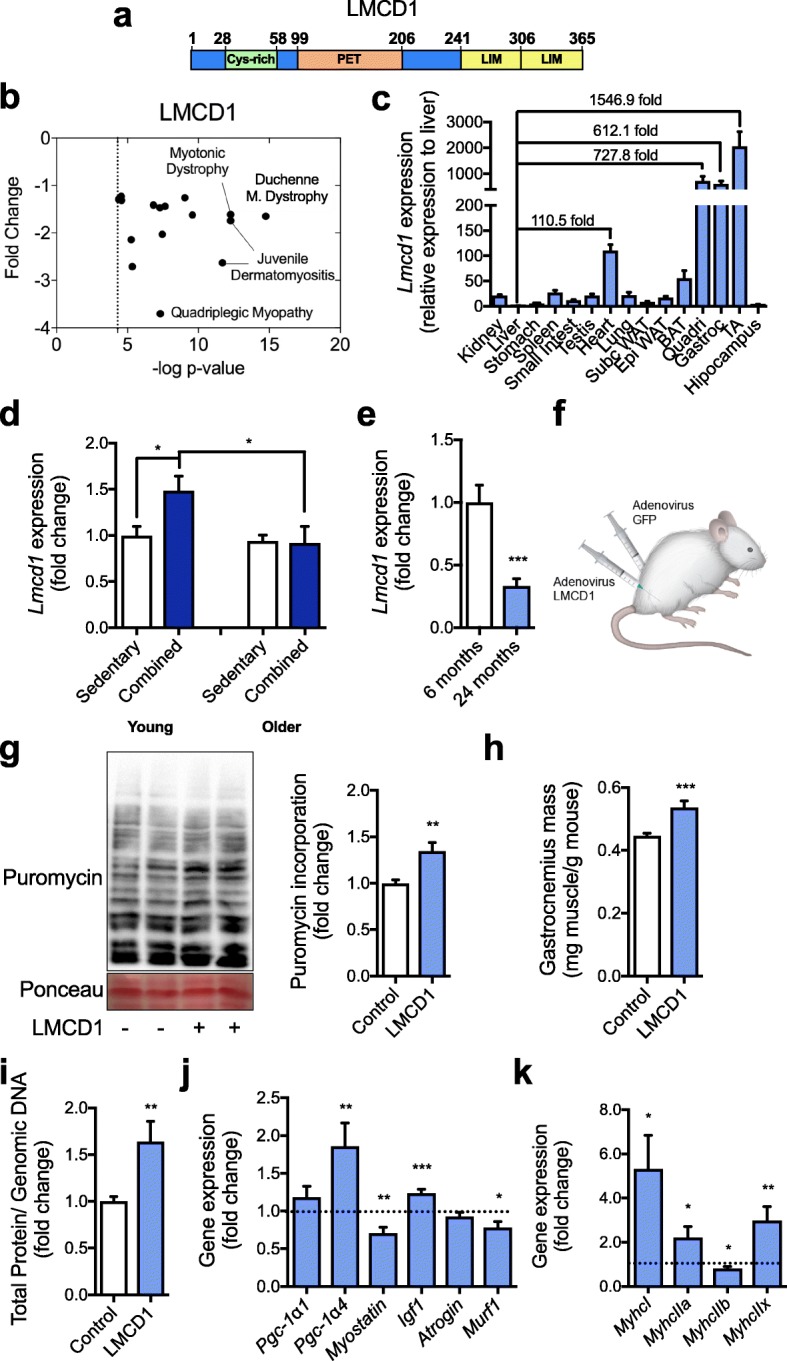
*Lmcd1* expression induces protein synthesis and muscle hypertrophy. **a** Schematic representation of the protein domain structure of LMCD1. Numbers indicate amino acid positions in mouse LMCD1. Cys-rich, cysteine-rich domain. PET, Prickle, Espinas, and Testin domain. LIM, Lin11, Isl-1, and Mec-3 domain. **b** Skeletal muscle *Lmcd1* mRNA expression in different available datasets of human diseases compared to healthy subjects. **c** Determination of *Lmcd1* mRNA levels in different mouse tissues by quantitative RT-PCR (qRT-PCR). Results are presented relative to the tissue with the lowest LMCD1 expression (liver) (*n* = 4). **d** qRT-PCR of *Lmcd1* mRNA levels in muscle biopsies from young and older human volunteers after 12 weeks of a combined exercised protocol vs sedentary controls. Results are presented relative to young sedentary human samples (*n* = 6). **e** *Lmcd1* mRNA levels by qRT-PCR in young (6 months) and older (24 months) mouse muscle. **f** Schematic representation of Green Fluorescent Protein (GFP) and LMCD1 adenovirus injection in each *gastrocnemius* from the hindlimb of 14-day-old SCID mice. **g** Muscle protein synthesis determined by puromycin incorporation followed by immunoblotting using specific antibody, and corresponding quantification (*n* = 6). **h** *Gastrocnemius* mass normalized by mouse body weight and **i** total protein/genomic DNA ratio after 7 days of GFP (control) or Lmcd1 expression (*n* = 6). **j** qRT-PCR of peroxisome proliferator-activated receptor gamma coactivator-1alpha1 (*Pgc-1α1*), *Pgc-1α4*, *myostatin*, insulin-like growth factor 1 (*Igf-1*), *atrogin*, and muscle RING-finger protein-1 (*Murf1*; *n* = 6). **k** qRT-PCR of myosin heavy chain (*Myhc*) *I*, *IIa*, *IIb*, and *IIx* (*n* = 6). Data is shown as mean ± SEM and *p < 0.05; **p < 0.01; ***p < 0.001

Here, we identify LIM and cysteine-rich domains 1 (LMCD1) as a novel player in the control of skeletal muscle mass. LMCD1 is part of a group of proteins initially defined by the zinc finger motifs found in Lin11, Isl-1, and Mec-3 (LIM). In addition to the two C-terminal LIM domains, LMCD1 contains a cysteine-rich domain in the amino-terminal region and a PET domain (named after Prickle, Espinas, and Testin) in the central part of the protein (Fig. 1a). Since these domains are often involved in protein-protein interactions, it is predicted that those are responsible for the actions of LMCD1. Through this mechanism LMCD1 represses *Gata6* actions in cultured cardiac cells [7] and interacts with calcineurin to induce cardiomyocyte hypertrophy [8, 9]. To date, there is no described function for LMCD1 in skeletal muscle.

High levels of Ca^2+^ lead to calcineurin activation by promoting its association with calmodulin. To facilitate calcineurin activation and skeletal muscle contraction/relaxation, the sarcoplasmic reticulum (SR) has one crucial transporter, the sarcoplasmic/endoplasmic reticulum calcium ATPase (SERCA). Recently, it was shown that SERCA is inhibited by direct interaction with a micropeptide known as myoregulin (MLN), that prevents Ca^2+^ uptake into the sarcoplasmic reticulum [10]. Here, we show that LMCD1 is a regulator of skeletal muscle mass and function that improves protein synthesis, force, and intracellular Ca^2+^ handling by regulating calcineurin activity and MLN expression.

## Methods

### Animal experiments

All animal experiments were performed according to the regional animal ethics committee of Northern Stockholm, which approved all experiments and protocols. CB-17/lcr-Prkdcscid/Rj (SCID) and C57Bl6/J mice (Janvier, France) were maintained on a standard rodent chow diet with 12 h light and dark cycles. Samples from young and older mice have been previously reported [11].

### Human muscle biopsies

Muscle samples from young and older human volunteers that underwent different exercise protocols have been previously reported [12]. *Lmcd1* mRNA expression in different available datasets of human diseases was compared with healthy subjects (Accession Numbers GSE3307, GSE7014, E-MEXP-2681, GSE10760, GSE1007, GSE11971, GSE39454).

### Primary myoblasts culture

Primary satellite cells (myoblasts) were isolated from C57Bl6/J as described previously [13]. Myoblasts were cultured in collagen-coated plates and maintained in Ham’s F-10/DMEM media mixture (Thermo Fisher Scientific) supplemented with 20% FBS (Sigma-Aldrich), 1% penicillin/streptomycin (Thermo Fisher Scientific), and 2.5 ng/mL basic fibroblast growth factor (Thermo Fisher Scientific). To induce differentiation into myotubes, cells were shifted to DMEM high glucose with pyruvate (Thermo Fisher Scientific) media supplemented with 5% horse serum (Thermo Fisher Scientific) and 1% penicillin/streptomycin (Thermo Fisher Scientific). Forty-eight hours after induction of differentiation, cells were transduced with an adenovirus to induce or inhibit the expression of *Lmcd1* or a control adenovirus. Twelve hours after transduction medium was replaced with fresh differentiation medium and approximately 48 h post-transduction, cells were harvested.

### Adenovirus-mediated expression

Adenovirus expressing green fluorescent protein (GFP) alone, and the ones expressing a control scrambled shRNA sequence were generated by using the pAdTrack/pAdEasy system (Stratagene), while adenovirus expression *Lmcd1* and sh*Lmcd1* were acquired from VectorBiolabs. Of note, all adenovirus express GFP (or mCherry, as indicated) from an independent promoter and *Lmcd1*, *shControl*, or sh*Lmcd1* from another. This allows us to monitor transduction efficiency without using fusion proteins. For cell culture experiments, differentiated myotubes were transduced with an adenovirus at a MOI of 100 for 12 h. For intramuscular delivery of adenovirus, the viral stock (2 μL/g of mice, 2 × 10^10^ infectious particles) was injected into the hind limbs or the hind paws of 14-day-old SCID mice. Each mouse received the control adenovirus (GFP or shControl) in one limb/paw and the LMCD1 or shLMCD1 in the contralateral limb/paw. Tissues were harvested and weighted 7-days after injection.

### Gene expression analysis

Total RNA was isolated using Isol-RNA Lysis Reagent (5 PRIME), according to manufacturer’s instructions. Afterward, 1 μg of RNA was treated with Amplification Grade DNase I (Life Technologies) and from that, 500 ng were used for cDNA preparation using the Applied Biosystem Reverse Transcription Kit (Life Technologies). Quantitative real-time PCR was performed in a ViiA 7 Real-Time PCR system thermal cycler with SYBR Green PCR Master Mix (both Applied Biosystems). Analysis of gene expression was performed using the ΔΔCt method and relative gene expression was normalized to hypoxanthine phosphoribosyltransferase (HPRT) mRNA levels. Additional file 1: Table S1 shows the primer sequences used for gene expression analysis.

### Total protein/genomic DNA ratio

Total protein and genomic DNA were obtained from the initial tube containing the interphase and phenol phase from the RNA extraction (Isol-RNA Lysis-chloroform fractions) from previous RNA extraction phase separation and preceded according to the manufacturer’s instructions. Briefly, 100% ethanol was added to precipitate DNA. Tubes were mixed by inversion and centrifuged at 2000 *g* for 2 min, at 4 °C. The phenol-ethanol supernatant was removed to 2 mL tubes, for protein extraction. DNA pellet was washed with 0.1 M sodium citrate in 10% ethanol with 30-min incubation at room temperature with mixing by inversion every 5 min. In between washes, samples were centrifuged at 2000 *g* for 5 min at 4 °C. Next, DNA pellets were washed with 75% ethanol with 20-min incubation at room temperature with mixing by inversion every 5 min followed by centrifugation at 2000 *g* for 5 min at 4 °C. After air-drying the pellet for 10 min, 8 mM of NaOH was added to re-dissolve the pellet. A final centrifugation was performed at 14,000 *g* for 10 min at room temperature to remove any insoluble material. Samples were neutralized with 0.1 M HEPES and 100 mM EDTA. DNA was quantified using the NanoDrop system (Thermo Fisher Scientifics).

Protein was precipitated from the phenol/ethanol supernatant with isopropanol, followed by mixing, and 10-min incubation at room temperature. Subsequently, samples were centrifuged at 12,000 *g* for 10 min at 4 °C and the supernatant discarded. Protein pellets were next washed three times with 0.3 M guanidine hydrochloride in 95% ethanol. In each wash, tubes were vigorously shaken, incubated at room temperature for 20 min followed by centrifugation at 7500 *g* for 5 min at room temperature. After the final wash and spin, 100% ethanol was added, and samples incubated at room temperature for 20 min, followed by a final centrifugation at 7500 *g* for 5 min at room temperature. The supernatant was removed, and the pellet air-dried for 10 min. After 10 M Urea with 50 mM DTT solution was added to the protein pellets, which were broken up with a needle. Then after 1-h incubation at room temperature, samples were heated up at 95 °C for 3 min and then placed on ice. While incubated on ice, samples were sonicated to help solubilizing the protein. Next, the samples were centrifuged at 10,000 *g* for 10 min at room temperature, to sediment insoluble material. Protein concentration was determined by Bradford Protein Assay following manufacturer’s instructions (BioRad).

### Puromycin incorporation

Cells were incubated with 15 μL of 10 μg/mL puromycin solution (SIGMA, diluted in PBS) for 10 min at 37 °C and 5% CO_2_. Cells were washed with pre-warmed media, new media was added, and cells were incubated for 50 min at 37 °C and 5% CO_2_. After that, cells were harvested for Western blot analysis. In vivo studies were performed by intraperitoneal injection of 0.040 μM puromycin in SCID mice for exactly 30 min. Gastrocnemius muscles were collected and snap frozen in liquid nitrogen for posterior Western blot analysis. Blots were incubated with anti-puromycin monoclonal antibody (12D10, Millipore). Each lane of the Western blot was normalized for the respective Ponceau staining and puromycin incorporation into protein is quantified as a measure of protein synthesis.

### Force and calcium measurements

Mice under isoflurane anesthesia were killed by cervical dislocation, and fast-twitch *flexor digitorum brevis* muscles were isolated by mechanical dissection and suspended between an adjustable hook and an Akers AE801 force transducer in the perfusion channel of a muscle bath placed on the stage of an inverted microscope [14]. The fiber was superfused at room temperature (25 °C) in Tyrode solution (in mM): NaCl, 121; KCl, 5.0; CaCl_2_, 1.8; MgCl_2_, 0.5; NaH_2_PO_4_, 0.4; NaHCO_3_, 24.0; EDTA, 0.1; glucose, 5.5; 0.2% fetal calf serum. The solution was bubbled with 95% O_2_–5% CO_2_. Fibers were stimulated with supramaximal current pulses with pulse duration of 0.5 ms delivered via platinum plate electrodes lying parallel to the fiber. Tetanic force was measured at 70 Hz force with 350 ms tetani at 2-s intervals for 50 contractions, where it was maximal and expressed relative to the fiber cross-sectional area.

Myoplasmic-free [Ca^2+^] ([Ca^2+^]_i_) was measured with the fluorescent Ca^2+^ indicator indo-1 AM (Molecular Probes/Invitrogen). Indo-1 AM was mixed in Tyrode buffer to a final concentration of 50 μM and added to the fiber for 30 min. The dye was excited with light at 360 ± 5 nm, and the light emitted at 405 ± 5 and 495 ± 5 nm was measured with two photomultiplier tubes. The 405/495 ratio (R) was translated to [Ca^2+^]_i_ using the following equation:

$$ {\left[{\mathrm{Ca}}^{2+}\right]}_{\mathrm{i}}={K}_{\mathrm{D}}.\upbeta .\left(R-{R}_{\mathrm{min}}\right).{\left({R}_{\mathrm{max}}-R\right)}^{-1} $$

where *K*_D_ is the apparent dissociation constant of the dye, β the ratio of the 495 nm signals at very low and saturating [Ca^2+^]_i_, and *R*_min_ and *R*_max_ the ratios at very low and saturating [Ca^2+^]_i_, respectively [15]. The [Ca^2+^]_i_–frequency and force–frequency relationships were obtained by stimulating fibers with 350-ms duration tetani at 15–150 Hz at 1-min intervals.

### Western blot

Muscle tissue and cell extracts were lysed in and total protein was extracted. Protein concentration was determined by Bradford Protein Assay following manufacturer’s instructions (BioRad). Further, 50 μg of proteins were separated by SDS polyacrylamide gel membranes (PAGE) and transferred to PVDF membranes. After blocking with 5% skim milk, blots were incubated overnight at 4 °C with primary antibodies diluted in 0.1% bovine serum albumin (BSA). After washing, membranes were incubated with horseradish peroxidase-conjugated secondary antibodies for 1 h at room temperature. Blots were visualized by enhanced chemiluminescence (GE Healthcare) and exposed on Chemidoc (BioRad). Quantification was performed using Image Lab software (BioRad) normalizing the values with α-Tubulin.

### Immunocytochemistry

Cultures of differentiated myotubes were washed with PBS and then fixed using 4% paraformaldehyde in PBS for 30 min at room temperature. Cultures were then washed again with PBS, and treated with PBS containing 0.1% Triton X-100 and 10% goat serum to block non-specific binding and facilitate cell permeabilization. Primary antibody (LMCD1; 1:200) was incubated with PBS containing 0.1% Triton X-100 and 10% goat serum at 4 °C overnight in a humidified chamber. We then washed cultures with PBS and incubated them with secondary antibody (Alexa Fluor 594 Goat Anti-Rabbit; 1:200) for 2 h at room temperature in a humidified chamber. Cell nuclei was stained with ProLong® Diamond Antifade with DAPI (Molecular Probes, 1:1000), which also contains a mounting agent. After 24 h, images were taken on a Nikon Inverted Microscope Eclipse TE300 and quantification of immunolabeling was performed using ImageJ.

### Cross-sectional area

Muscles were collected, coated in OCT, and frozen in pre-cooled isopentane to prevent freeze-damage. Then, 10-μm-thick sections of the gastrocnemius muscle were stained either with Hematoxylin & Eosin or wheat germ agglutinin Texas Red™-X Conjugate (ThermoFisher) to detect lectins from the sarcolemma and estimate fiber size and cross-sectional area of the muscles. Three animals per group were used and several images were taken to cover the complete sectioning area.

### Myoblast fusion index

Forty-eight hours post-transduction with *GFP* control or *Lmcd1* adenovirus, cells were fixed with 4% paraformaldehyde for 10 min. Cell nuclei were stained with ProLong® Diamond Antifade with DAPI (Molecular Probes, 1:1000), which also contains a mounting agent. After 24 h, images were taken on a Zeiss Inverted Microscope and quantification of nuclei was performed using ImageJ. The fusion index was determined as the ratio between the number of nuclei inside a myotube and the total number of nuclei in the image.

### Cyclosporine A treatment

After adenovirus injections either in the gastrocnemius or *flexor digitorum brevis* (FDB), SCID mice were injected daily intraperitoneally for 7 days with 25 mg cyclosporine A (CsA)/kg mouse to inhibit calcineurin. FDB was collected for force and calcium measurements and gastrocnemius was collected for gene expression. Alternatively, mice were also injected intraperitoneally with 0.040 μM puromycin for 30 min to measure protein synthesis.

### Luciferase assays

C2C12 cells were transfected with a total of 1 μg of luciferase plasmid DNA together with scrambled siRNA or siCalcinuerin (Thermo Fisher) using Lipofectamine 2000 (Thermo Fisher) 24 h after seeding. After overnight transfection, differentiation media was added to start differentiation. After 2 days, cells where transduced with GFP (control) or LMCD1 adenovirus. Two days after transduction, cells in each well were lysed in 200 μL of passive lysis buffer (Promega) and subjected to a single freeze–thaw cycle at − 80 °C. Luciferase activity was measured for each well by reacting 10 μL of cell lysate with 25 μL of luciferase assay buffer (Promega) in white 96-well plates, using a microplate reader. Individual wells were normalized to Renila activity using the Dual-Luciferase Kit (Promega).

### Kinase activity assay with PamChip®

Cell lysates were obtained using M-PER buffer containing phosphatase and protease inhibitors (all from Thermo Scientific). Total lysate (5 μg) was applied per well on the PamChip**®**. Meanwhile, a tyrosine peptide kinase PamChip**®** (Pamgene) was blocked with 2% bovine serum albumin using the standard setting of a PamStation**®** 12 System (Pamgene). The composition of the kinase buffer was as follows: 1× PK buffer, 1× PTK additive, 10 mM DTT, 0.5 mg/mL bovine serum albumin, 400 μM ATP, and 5 μg/mL PY20-FITC. The kinase buffer was prepared and chilled on ice, and the solution is added to 5 μg ice-cold lysates and immediately applied to the tyrosine peptide kinase PamChip**®**. The assay was run on a PamStation® 12 System according to standard procedures. For the serine/threonine peptide kinase PamChip**®** (Pamgene), the PamChip**®** was blocked with 2% bovine serum albumin using the standard setting of a PamStation® 12 System (Pamgene). The composition of the kinase buffer was as follows: 1× PK buffer, 1× STK antibody mix, 0.5 mg/mL bovine serum albumin, and 400 μM ATP. The kinase buffer was prepared and chilled on ice, and the solution is added to 5 μg ice-cold lysates and immediately applied to the tyrosine peptide kinase PamChip**®**. The assay was run on a PamStation® 12 System according to standard procedures. After incubation, detection mix (1× antibody buffer, 5 μg/mL STK antibody FITC-labeled) was prepared and added to the wells. Detection was run on a PamStation® 12 System according to standard procedures.

The data (spot images) were further quantified and linked to the peptide identities using BioNavigator® software. The quantified data were then fitted to a curve using CurveFitHT® software using the Vinip2 curve fitting algorithm; the initial velocity is derived at the first kinetic time point read. Replicates from visually damaged wells or with interreplicate correlation coefficients lower than 0.95 were removed from the analysis. All subsequent data analyses were done on initial velocities (*V*_ini_) using R software packages. Each peptide was assigned a score based on difference of the mean signal of the peptide divided by the estimation of the variation of the peptide between control and experimental condition. PhoshphoNET database from Kinexus was queried to retrieve top eight kinases for each peptide. Kinase score was calculated as an average of the scores for each associated peptide in its set. Significance scores were calculated by permutation of peptide labels and is determined by the formula *Q* = -10log[max(*m*/*M*,1/*M*)], where *m* is the number of times out of *M* permutations where calculated permuted score was higher than the original calculated value without permutation. Top 35 kinases based on the kinase score were submitted to string-db.org to cluster the kinases and identify possible regulated pathways. Out of the different functional enrichments reported by string-db.org, KEGG pathway database, Reactome pathway database, and Uniprot keywords were plotted to determine processes regulated by the top 35 kinases. Overrepresentation scores are plotted as -log10(*p* value).

### Statistical analysis

All results are expressed as means ± SD for cell experiments and ± SEM for animal experiments. Statistical analysis was performed using GraphPad Prism 6. Two-tailed Student’s t test and two-way ANOVA and post-hoc analysis for the force and calcium measurements were used to determine *p* values. Statistical significance was defined as *p* < 0.05.

## Results

### *Lmcd1* expression is high in skeletal muscle but decreases with disease and aging

To identify novel potential regulators of skeletal muscle mass and strength, we analyzed gene expression data from muscle of patients with diverse muscle pathologies (Fig. 1b). From this analysis, we identified *Lmcd1* (Fig. 1a), which showed significantly decreased expression in diseases such as Duchenne muscular dystrophy and myotonic dystrophy (Fig. 1b). To determine how the skeletal muscle expression levels of LMCD1 compare with other organs, we screened a panel of tissues from 2-month-old mice by qRT-PCR. Results are shown as expression relative to the liver, the tissue with the lowest *Lmcd1* mRNA levels (Fig. 1c). We observed that although *Lmcd1* is robustly expressed in the heart (110.5-fold vs. liver), its highest expression is in skeletal muscle (727.8- and 612.1-fold in quadriceps and *gastrocnemius*, respectively, and 1546.9-fold in *tibialis anterioris*—TA). This is in agreement with what has been observed in human tissues by Northern blotting [16]. We could also observe significant *Lmcd1* expression in brown adipose tissue but low levels in white adipose depots (Fig. 1c). Although there are no differences at sedentary levels, after combined resistance and endurance exercise training [12], LMCD1 expression increases in human skeletal muscle. However, this effect is abrogated in older subjects (18–30 vs. 65–80 years old; Fig. 1d). On the other hand, older mice (24 months vs. 6 months [11]; showed significantly decreased *Lmcd1* expression in skeletal muscle (Fig. 1e), which was not observed in older human subjects (Fig. 1d). Interestingly, in a skeletal muscle gene expression data set obtained from tumor-bearing mice, *LMCD1* expression decreases during the first weeks of cancer cachexia [17].

### LMCD1 induces skeletal muscle hypertrophy in vivo

To assess if increasing LMCD1 levels is sufficient to induce changes in skeletal muscle mass, we transiently expressed it in mouse *gastrocnemius* muscle. To this end, a control adenoviral vector (expressing green fluorescent protein, GFP) was delivered to one limb, while the contralateral muscle was transduced with an adenovirus expressing *Lmcd1* and GFP from two separate and independent promoters, which allows to determine transduction efficiency without using fusion proteins (Fig. 1f). Within 7-days post-injection, we observed an increase in protein synthesis determined by puromycin incorporation into neo-synthesized proteins, compared to the GFP-injected contralateral *gastrocnemius* (Fig. 1g). Ectopic *Lmcd1* expression resulted in an increase in the *gastrocnemius* muscle mass (Fig. 1h). This was also translated in an increase in the ratio of total protein/genomic DNA and in a modest increase in the percentage of fibers with higher cross-sectional area, compared to the control, GFP-transduced *gastrocnemius* (Fig. 1i and Additional file 3: Figure S1a). Altogether, those results indicate that LMCD1 induces hypertrophy with an increase in protein synthesis. We next evaluated if these changes were reflected in the expression of genes related to skeletal muscle mass maintenance. In line with the observed LMCD1-induced hypertrophy, we did observe a modest induction of *Pgc*-*1α4* and *Igf*-*1* expression and a decrease in myostatin and muscle RING-finger protein-1 (*Murf1*) mRNA levels (Fig. 1j). In addition, *Lmcd1* overexpression increased the expression of myosin heavy chain (*Myhc*) type I, IIa, and IIx with a decrease in IIb (Fig. 1k). However, the magnitude of these effects suggests that these changes in gene expression might be consequence of signaling pathways activated by LMCD1, as opposed to direct effects on gene transcription. No major changes were observed in the expression of genes related to SR-stress, thick and thin filaments, and Z-disc proteins, after LMCD1 overexpression (Additional file 3: Figure S1b–e).

### LMCD1 increases specific force with less SR Ca^2+^ release, and resistance to fatigue

**Fig. 2:**
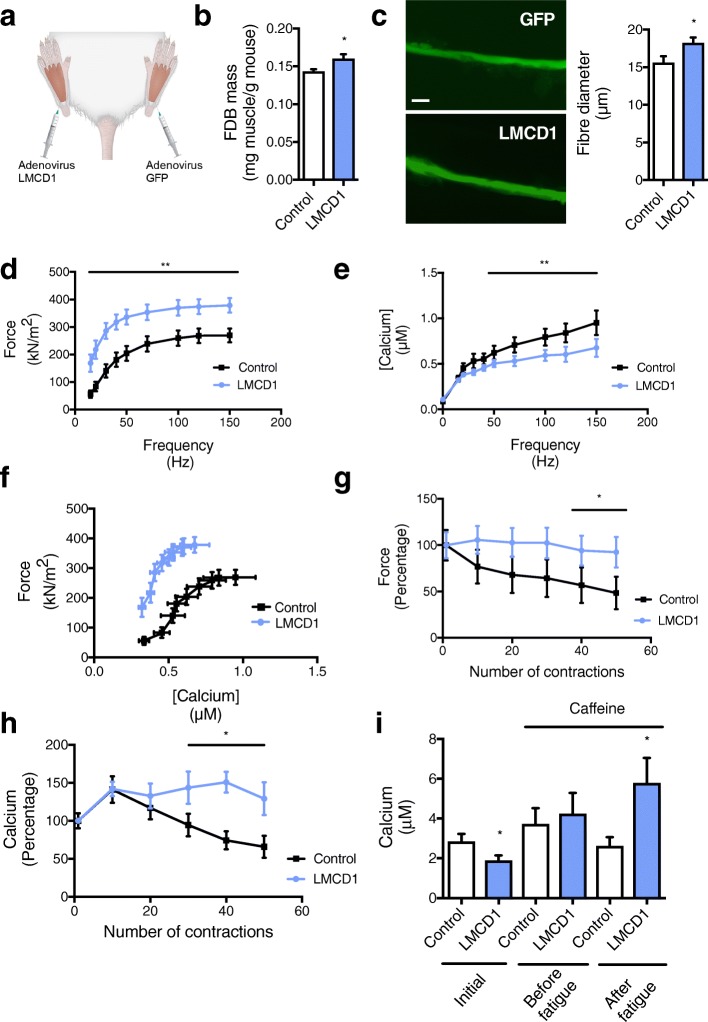
LMCD1 increases fiber diameter, force, and resistance to fatigue. **a** Schematic representation of Green Fluorescent Protein (GFP) and LMCD1 adenovirus injection in each *flexor digitorum brevis* (FDB) muscle from 14-day-old SCID mice. **b** FDB mass normalized by mouse body weight after 7 days of GFP (control) or Lmcd1 expression (*n* = 6). **c** Isolated fiber diameter and representative microscopy image (× 200) in mice treated as in (**b**) (*n* = 10 mice, 15–18 fibers). Scale bar = 30 μm. **d** Force normalized by cross-sectional area (specific force) for the different frequencies tested (15–150 Hz) at 1-min intervals in mice treated as in (**b**) (*n* = 10 mice, 15–18 fibers). **e** Myoplasmic free Ca^2+^ concentrations for the different frequencies tested (15–150 Hz) at 1-min intervals in mice treated as in (**b**) (*n* = 10 mice, 15–18 fibers). **f** Mean value for specific force and myoplasmic free Ca^2+^ concentration for the different frequencies tested (15–150 Hz) at 1-min intervals, in mice treated as in (**b**) (*n* = 10 mice, 15–18 fibers). **g** Percentage of force relative to the first contraction. Peak force was measured at 70 Hz tetani of 350-ms duration given at 2-s intervals for 50 contractions in mice treated as in (**b**) (*n* = 10 mice, 15–18 fibers). **h** Percentage of myoplasmic free Ca^2+^ concentration normalized for the first contraction. Myoplasmic free Ca^2+^ concentration was measured as in (**g**) (*n* = 10 mice, 15–18 fibers). **i** Myoplasmic free Ca^2+^ concentrations at 150 Hz with caffeine treatment before and after fatigue protocol as in (**g**) (*n* = 10). Data is shown as mean ± SEM and *p < 0.05; **p < 0.01

To assess if skeletal muscle hypertrophy induced by LMCD1 was translated into an increase in force, we transduced the *flexor digitorum brevis* (FDB) muscle of one mouse hind paw with the control GFP adenovirus and the contralateral muscle with the LMCD1 adenovirus (Fig. 2a). Seven days post-injection, FDB muscles overexpressing *Lmcd1* showed an increase in muscle mass and diameter of individual fibers (Fig. 2b–c), compared with the contralateral GFP-transduced FDB muscle. Remarkably, when measuring specific force (absolute force normalized by the cross-sectional area of the fiber) and SR Ca^2+^ release in individual fibers, *Lmcd1* overexpression induced an increase in specific force with less SR Ca^2+^ release compared with GFP-control muscle for the different frequencies analyzed (Fig. 2d–f). Interestingly, when measuring fatigue, *Lmcd1* overexpression showed fatigue resistance with no decline in force or SR Ca^2+^ release after 50 contractions (Fig. 2g–h). To confirm that the levels of SR Ca^2+^ are not different from control conditions, we performed a caffeine treatment before and after the fatigue protocol (Fig. 2i). Caffeine treatment is often used to determine the pool of Ca^2+^ in the SR since it induces its release. SR Ca^2+^ did not change with *Lmcd1* overexpression but increased after a fatigue protocol, suggesting that LMCD1 has a protective effect against fatigue in skeletal muscle fibers by increasing the reuptake of SR Ca^2+^.

### In vivo *Lmcd1* silencing impairs SR Ca^2+^ handling and decreases force but does not reduce skeletal muscle mass

**Fig. 3:**
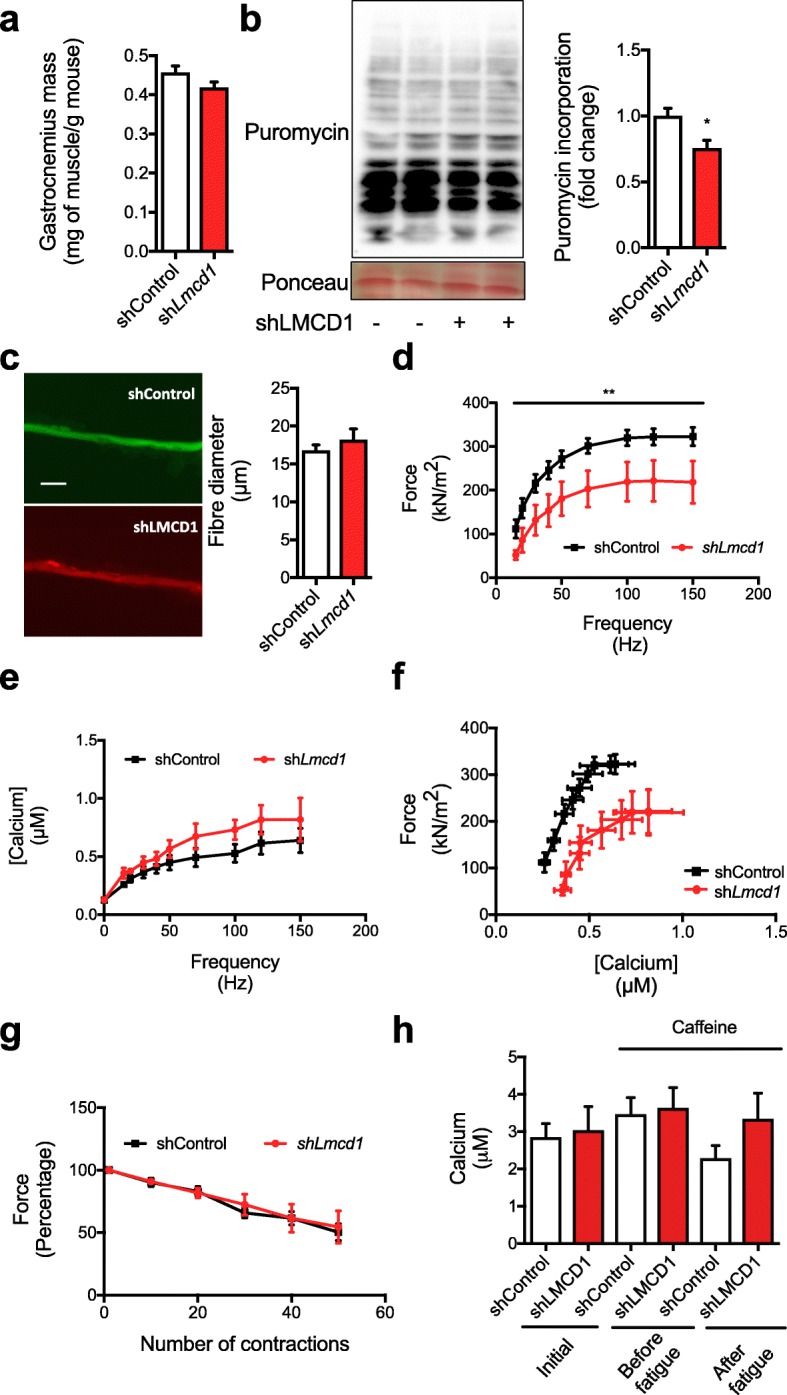
Lmcd1 silencing results in reduced protein synthesis and decreased intrinsic force, without muscle atrophy. **a** *Gastrocnemius* mass normalized by mouse body weight 7 days after intramuscular delivery of a scrambled/control shRNA or a sh*Lmcd1* (*n* = 6). **b** In vivo muscle protein synthesis by puromycin incorporation, and corresponding quantification (*n* = 6). **c** Isolated fiber diameter and representative microscopy image (× 200) in mice treated as in (**a**) (*n* = 10). Scale bar = 30 μm. **d** Force normalized for the cross-sectional area (specific force) for the different frequency tested (15–150 Hz) at 1-min interval in mice treated as in (**a**) (*n* = 10). **e** Myoplasmic free Ca^2+^ concentration for the different frequency tested (15–150 Hz) at 1-min interval in mice treated as in (**a**) (*n* = 10). **f** Mean value for specific force and myoplasmic free Ca^2+^ concentration for the different frequencies tested (15–150 Hz) at 1 min intervals in mice treated as in (**a**) (*n* = 10). **g** Percentage of force relative to the first contraction. Peak force was measured at 70 Hz tetani of 350 ms duration given at 2-s intervals for 50 contractions in mice treated as in (**a**) (*n* = 10). **h** Myoplasmic free Ca^2+^ concentrations at 150 Hz with caffeine treatment before and after fatigue protocol as in (**g**) (*n* = 10). Data is shown as mean ± SEM and *p < 0.05; **p < 0.01

To evaluate if loss of LMCD1 expression resulted in skeletal muscle atrophy and reduced force, we performed loss-of-function experiments using an adenoviral-encoded shRNA against *Lmcd1*, which allowed an almost 60% reduction in *LMCD1* levels (Additional file 3: Figure S2a). Although we did not observe significant differences in the *gastrocnemius* mass upon *Lmcd1* silencing (Fig. 3a), we could still determine a decrease in protein synthesis/puromycin incorporation into newly synthesized proteins when compared to a scrambled shRNA control (Fig. 3b). For functional measurements, we used isolated FDB fibers with *Lmcd1* knockdown vs. control (as depicted in Fig. 2a). Interestingly, despite no changes in FDB fiber diameter upon *Lmcd1* silencing (Fig. 3c), those fibers produced lower specific force without significant changes in SR Ca^2+^ release (compared to the scrambled shRNA control fibers) (Fig. 3d–f). The fatigue phenotype was also similar to control (Fig. 3g). In fact, specific force is lower in sh*Lmcd1* fibers than in shRNA control fibers (Fig. 3d), suggesting the importance of LMCD1 for force maintenance. In addition, caffeine treatment before and after the fatigue protocol (Fig. 3h), showed no changes in SR Ca^2+^ upon *Lmcd1* silencing. These results confirmed the crucial effect of LMCD1 in force and Ca^2+^ handling together with fatigue resistance.

### LMCD1-induced kinase activity profiling in mouse primary myotubes

**Fig. 4:**
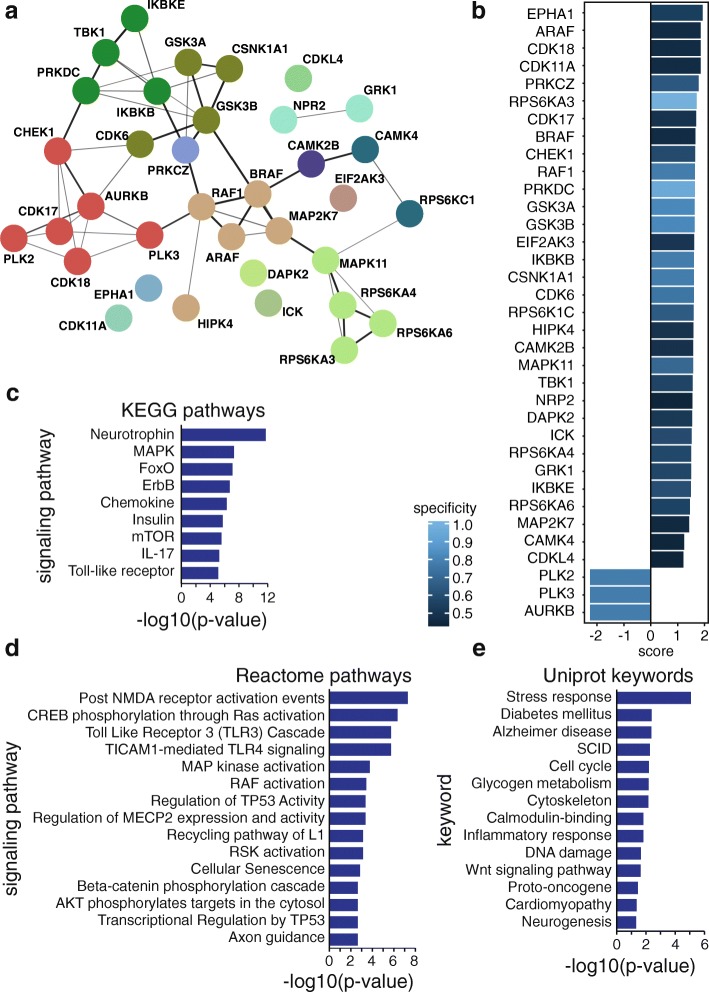
*Lmcd1* overexpression alters several kinase activities in mouse primary myotubes related to skeletal muscle function. **a** Top 35 kinases interactions that significantly changed in a kinase activity assay in differentiated primary mouse myotubes after 2 days transduction with GFP (control) or LMCD1 adenovirus (*n* = 4). **b** Top 35 kinases scores, taking into account specificity and selectivity, that significantly changed in a kinase activity assay in myotubes transduced as in (**a**) (*n* = 4). **c** Top Kyoto Encyclopedia of Genes and Genomes (KEGG) signaling pathways of the top kinases that significantly changed in a kinase activity assay in myotubes transduced as in (**a**) (*n* = 4). **d** Top Reactome signaling pathways of the top kinases that significantly changed in a kinase activity assay in myotubes transduced as in (**a**) (*n* = 4). **e** Top Uniprot keywords of the top kinases that significantly changed in a kinase activity assay in myotubes transduced as in (**a**) (*n* = 4). *EPHA1* Ephrin type-A receptor 1, *CDK18* cyclin-dependent kinase 18, *CDK11A* cyclin-dependent kinase 11A, *PRKCZ* protein kinase C zeta, *RPS6KA3* ribosomal protein S6 kinase A3, *CDK17* cyclin-dependent kinase 17, *CHEK1* checkpoint kinase 1, *PRKDC* protein kinase DNA-activated catalytic subunit, *GSK3A* glycogen synthase kinase-3 alpha, *GSK3B* glycogen synthase kinase-3 beta, *EIF2AK3* eukaryotic translation initiation factor 2-alpha kinase 3, *IKBKB* inhibitor of nuclear factor kappa B kinase subunit beta, *CSNK1A1* casein kinase 1 alpha 1, *CDK6* cyclin-dependent 6, *RPS6KC1* ribosomal protein S6 kinase C1, *HIPK4* homeodomain interacting protein kinase 4, *CAMK2B* calcium/calmodulin-dependent protein kinase II beta, *MAPK11* mitogen-activated protein kinase 11, *TBK1* tumor necrosis factor receptor-associated factor binding kinase 1, *NRP2* neuropilin 2, *DAPK2* death associated protein kinase 2, *ICK* intestinal cell kinase, *RPS6KA4* ribosomal protein S6 kinase A4, *GRK1* G protein-coupled receptor kinase 1, *IKBKE* inhibitor of nuclear factor kappa B kinase subunit epsilon, *RPS6KA6* ribosomal protein S6 kinase A6, *MAP2* K7 dual specificity mitogen-activated protein kinase 7, *CAMK4* calcium/calmodulin-dependent protein kinase type IV, *CDKL4* cyclin-dependent kinase Like 4, *PLK2* polo like kinase 2, *PLK3* polo like kinase 3, *AURKB* aurora kinase B, *FoxO* forkhead box, *mTOR* mammalian target of rapamycin

To determine the molecular mechanism by which LMCD1 increases muscle hypertrophy, increased force, and calcium handling, we used mouse primary myotube cultures. To increase the levels of LMCD1, fully differentiated myotubes were transduced as above (i.e., LMCD1 vs GFP adenovirus). To understand the molecular effects of LMCD1, general kinase activities were evaluated using PamChip® kinase activity arrays. Following phosphorylation patterns of the peptides, we could determine that several kinase activities were altered in the presence of LMCD1 overexpression. Interestingly, when consolidating the top 35 kinases, we could determine that some were involved in protein synthesis (RPS6KA3, RPS6KA4, RPS6KA6, RPS6KC1) and calcium signaling (CAMK2B, CAMK4). These results confirmed our previous findings of increased protein synthesis and calcium handling (Fig. 4a, b and Additional file 2: Table S2). To better visualize LMCD1 signaling pathways, we used the Kyoto Encyclopedia of Genes and Genomes (KEGG), a database resource for understanding high-level functions in biological systems. Some of the top pathways were the mitogen-activated protein kinases (MAPKs) and the mammalian target of rapamycin (mTOR) (Fig. 4c), which are closely involved in protein synthesis and regulation of S6 kinase [18, 19]. To visually represent biological pathways in full mechanistic detail, we took into account the reactome signaling pathways (Fig. 4d). This software allowed us to confirm the regulation of S6 kinase in the KEGG signaling pathways by presenting also MAP kinase activation and AKT targets phosphorylation. In addition, one of the top signaling pathways presented by the reactome analysis was the cAMP-response-element-binding protein (CREB) phosphorylation. CREB activation occurs, for example, after calcium and β-adrenergic stimulation and it has been shown to induce anabolic changes that drive myofiber hypertrophy in vivo and in vitro [20]. Finally, we also organized the top kinases accordingly with the Uniprot Keywords, which can be used to retrieve subsets of protein entries or to generate indexes of entries based on functional, structural, or other categories (Fig. 4e). Using this approach, we could determine that the top kinases are involved, for instance, in cytoskeleton remodeling and calmodulin-binding. Since these kinase pathways could suggest that LMCD1 affects myoblast fusion, and although all experiments were performed in fully differentiated myotubes, we determined the myoblast fusion index, but no changes were observed between the two groups (GFP–75.9 ± 6.5%; LMCD1–74.7 ± 7.6%). Altogether, the kinase activity assay indicated that LMCD1 changes kinase activities that are responsible for the effects observed of increased protein synthesis, force and calcium handling.

### LMCD1 regulates protein synthesis in mouse primary myotubes

**Fig. 5:**
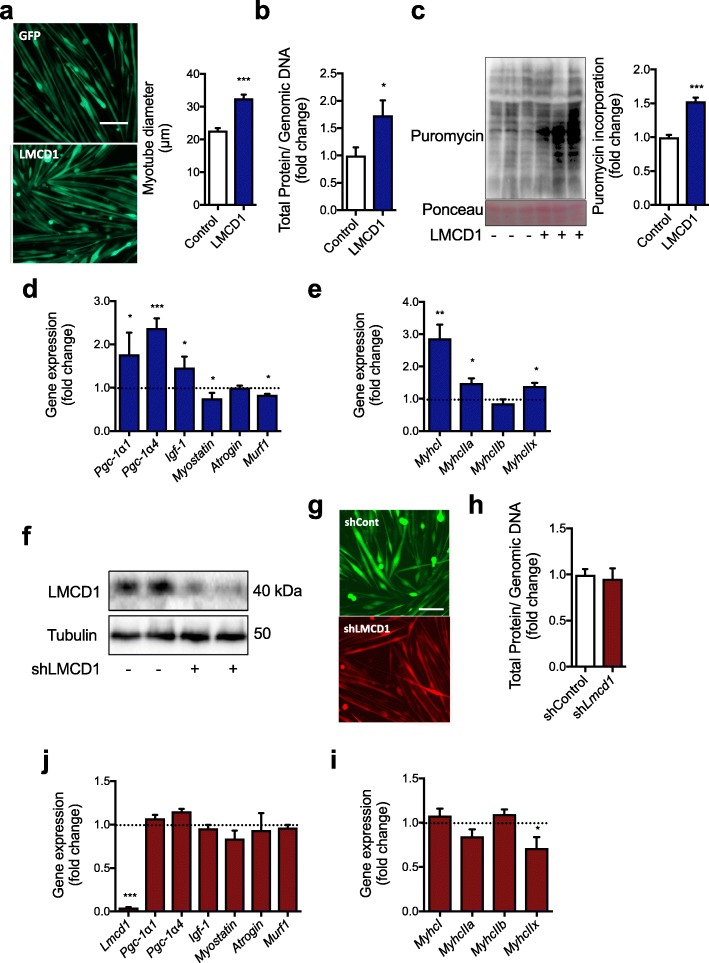
*Lmcd1* induces protein synthesis and myotube hypertrophy in mouse primary myotubes. **a** Representative microscopy image of differentiated mouse primary myotubes 2 days after transduction with GFP (control) or LMCD1 adenovirus (× 200; *n* = 8; scale bar = 100 μm) and corresponding myotube diameter measurements. **b** Total protein/genomic DNA ratio determined in myotubes transduced as in (**a**) (*n* = 8). **c** Protein synthesis determination by puromycin incorporation in differentiated primary mouse myotubes transduced as in (**a**), and respective quantification (*n* = 8). **d** qRT-PCR determination of peroxisome proliferator-activated receptor gamma coactivator-1alpha1 (*Pgc*-*1α1*), *Pgc*-*1α4*, *myostatin*, insulin-like growth factor 1 (*Igf*-*1*), *atrogin*, and muscle RING-finger protein-1 (*Murf1*; *n* = 8) in differentiated primary mouse myotubes transduced as in (**a**). **e** qRT-PCR of myosin heavy chain (*Myhc*) *I*, *IIa*, *IIb*, and *IIx* (*n* = 8) in differentiated primary mouse myotubes transduced as in (**a**). **f** Western blot of LMCD1 protein expression in differentiated primary mouse myotubes after 2 days transduction of scrambled shRNA or sh*Lmcd1* adenovirus (*n* = 8). **g** Representative microscopy image of differentiated mouse primary myotubes (× 200; *n* = 8; scale bar = 100 μm) transduced as in (**f**). **h** Total protein/genomic DNA ratio in differentiated primary mouse myotubes transduced as in (**f**) (*n* = 8). **j** qRT-PCR for *Lmcd1*, *Pgc*-*1α1*, *Pgc*-*1α4*, *myostatin*, *Igf*-*1*, *atrogin*, and *Murf1* (*n* = 8) in differentiated primary mouse myotubes transduced as in (**f**). **i** qRT-PCR of *MyhcI*, *IIa*, *IIb*, and *IIx* (*n* = 8) in differentiated primary mouse myotubes transduced as in (**f**). Data is shown as mean ± SD and *p < 0.05; **p < 0.01; ***p < 0.001

To confirm the mechanistic aspects of LMCD1 action determined by the kinase activity assay, we first confirmed whether we could obtain the same effects in vitro as we observed in vivo regarding hypertrophy and protein synthesis. For that, we used fully differentiated myotubes transduced with LMCD1 or GFP adenovirus. As seen in vivo, *Lmcd1* overexpression resulted in an increase in myotube size (approx. 40%) (Fig. 5a). We also observed an increase in the ratio of total protein/genomic DNA (Fig. 5b), and in puromycin incorporation into newly synthesized proteins (Fig. 5c), when compared to control myotubes. In agreement with our in vivo results (Fig. 1i), LMCD1 induced a slight increase in myotube *Pgc*-*1α4* and *Igf*-*1* expression, and a decrease in myostatin and *Murf1* mRNA levels (Fig. 5d). In addition, we could also determine an increase in the expression of *Myhc* type I, IIa, and IIx with a decrease in IIb expression (Fig. 5e). When we transduced differentiated mouse primary myotubes with the *Lmcd1* shRNA adenovirus, we achieved almost 70% reduction in LMCD1 protein levels (Fig. 5f). Despite this decrease in LMCD1 levels, we did not observe any differences in myotube size or total protein/genomic DNA ratio (Fig. 5g–h), confirming the same results seen in vivo (Fig. 3a–b). Moreover, *Lmcd1* silencing did not change the expression of several skeletal muscle genes (Fig. 5j and Additional file 3: Figure S2b–e). LMCD1 silencing led to a decrease in *MyhcIIx* expression, without changes in the other *Myhc* types (Fig. 5i). In addition, some of the genes involved in the regulation of SR stress were also decreased after LMCD1 silencing (Additional file 3: Figure S2b).

### LMCD1 regulates AKT/S6K signaling and myotube hypertrophy independently of 4E-BP1

**Fig. 6:**
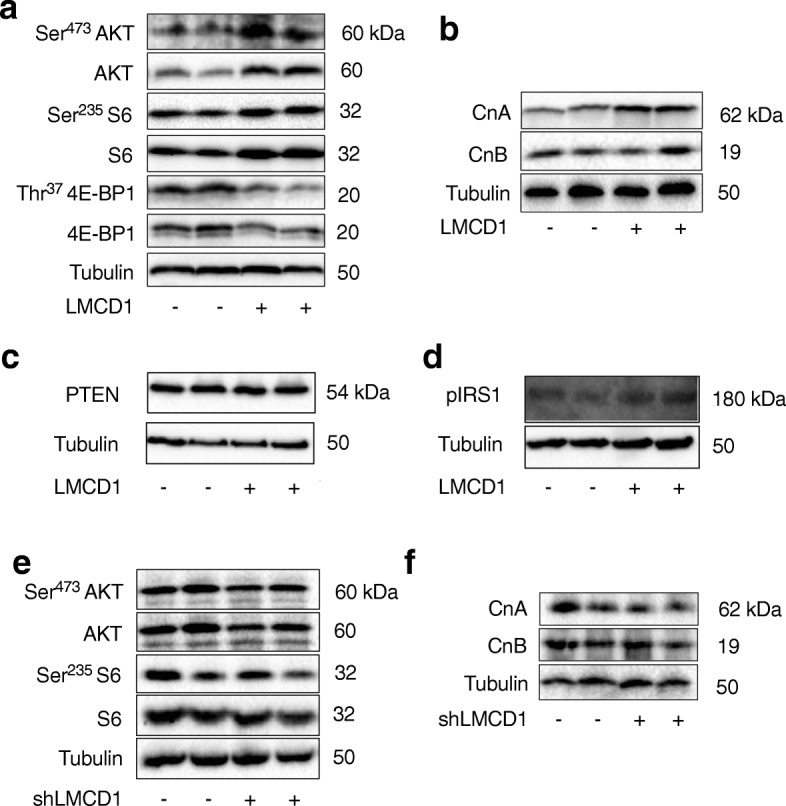
*Lmcd1* increases S6 and AKT phosphorylation as well as calcineurin expression while silencing of *Lmcd1* has the opposite effect. **a** Representative western blot for AKT Ser^473^ phosphorylation, total AKT, S6 Ser^235^ phosphorylation, total S6, 4E-BP1 Thr^37^phosphorylation, and total 4E-BP1, normalized for α-tubulin expression, in differentiated primary mouse myotubes 2 days after transduction with GFP (control) or LMCD1 adenovirus (*n* = 8). **b** Representative Western blot for calcineurin A and calcineurin B, normalized for α-tubulin expression, in differentiated myotubes transduced as in (**a**) (*n* = 8). **c** Representative western blot for PTEN normalized for α-tubulin expression in differentiated myotubes transduced as in (**a**) (*n* = 6). **d** Representative Western blot for insulin receptor substrate 1 (IRS1) Tyr^612^ phosphorylation normalized for α-tubulin expression in differentiated myotubes transduced as in (**a**) (*n* = 4). **e** Representative Western blot for AKT phosphorylation Ser^473^, total AKT, S6 phosphorylation Ser^235^, and total S6, normalized for α-tubulin expression, in differentiated primary mouse myotubes after 2 days transduction of scrambled shRNA or sh*Lmcd1* adenovirus (*n* = 8). **f** Representative western blot for calcineurin A and calcineurin B, normalized for α-tubulin expression, in differentiated primary mouse myotubes transduced as in (**e**) (*n* = 8)

Since the effects of increased protein synthesis and hypertrophy were observed in vivo and in vitro, next we determined if changes in S6 kinase, AKT phosphorylation, and calcium signaling were present in fully differentiated myotubes transduced either with LMCD1 or GFP adenovirus, as indicated by the kinase activity assay (Fig. 4). When we overexpressed LMCD1 in mouse primary myotubes, it increased the total protein and the phosphorylation levels of AKT and S6 (1.6-, 1.4-fold expression for total levels, respectively, Fig. 6a and Additional file 3: Figure S3a). This was also in agreement with our kinase activity assay (Fig. 4), that showed an increase in the S6 and AKT signaling. On the other hand, LMCD1 reduced total and phosphorylated levels of 4E-binding protein 1 (4E-BP1) (0.7-fold expression for total levels, Fig. 6a and Additional file 3: Figure S3a). These results support an increase in protein synthesis, which was already indicated by the kinase activity assay (Fig. 4), the determination of puromycin incorporation into newly synthetized proteins and increased total protein/genomic DNA ratio. Since LMCD1 improved Ca^2+^ handling, next we checked the Ca^2+^ signaling pathway. LMCD1 expression increased calcineurin A and B protein levels (1.4- and 1.2-fold, respectively), with slight changes in the expression of Ca^2+^ regulators or channel genes, specially the Na^+^/Ca^2+^ and Na^+^/K^+^/Ca^2+^ exchangers (Fig. 6b and Additional file 3: Figure S3a–c). Our next aim was to connect the increased protein synthesis and the Ca^2+^ signaling pathway. Indeed, calcineurin activation can lead to PTEN-mediated AKT dephosphorylation. Since P-Ser^473^AKT levels are increased in the presence of LMCD1, we determined PTEN levels but saw no significant changes in its protein levels (Fig. 6c and Additional file 3: Figure S3d). The other link between calcineurin and AKT activation is through the IGF1/IRS1 (insulin receptor substrate 1) axis, which can induce the phosphorylation of AKT, mTOR and finally protein synthesis. Accordingly, after *LMCD1* expression, we observed an increase in mTOR signaling (Fig. 4c) and in IRS1 phosphorylation (Fig. 6d and Additional file 3: Figure S3e), indicating that this might be the molecular connection between increased calcineurin and protein synthesis. This was also corroborated by our kinase activity assay, that showed an increase in insulin signaling-related activities (Fig. 4d).

Since we observed small effects on gene expression, our next step was to determine the cellular localization of LMCD1. For that, we performed immunochemistry in differentiated mouse primary myotubes. As expected, LMCD1 has a predominantly cytosolic localization in myotubes (Additional file 3: Figure S3f). In addition, *Lmcd1* overexpression in differentiated mouse primary myotubes did not induce any major changes in several skeletal muscle-related genes (Additional file 3: Figure S3 g–k), suggesting it acts primarily through cellular signaling mechanisms (as opposed to direct regulation of gene expression).

Interestingly, after silencing *Lmcd1*, there was a decrease in S6 and AKT total and phosphorylation (0.9-, 0.7-fold expression for total levels, respectively, Fig. 6e and Additional file 3: Figure S3 l) as well as in the levels of calcineurin A and B (Fig. 6f and Additional file 3: Figure S3 l), suggesting that *Lmcd1* silencing induces a decrease in the rate of protein synthesis and Ca^2+^ handling which reduces force but is not enough to reduce the muscle/myotube size. By reducing protein synthesis and Ca^2+^, the reduction in LMCD1 might contribute to the decrease in genes related to SR stress.

### Calcineurin inhibition impairs LMCD1 activity in vivo

**Fig. 7:**
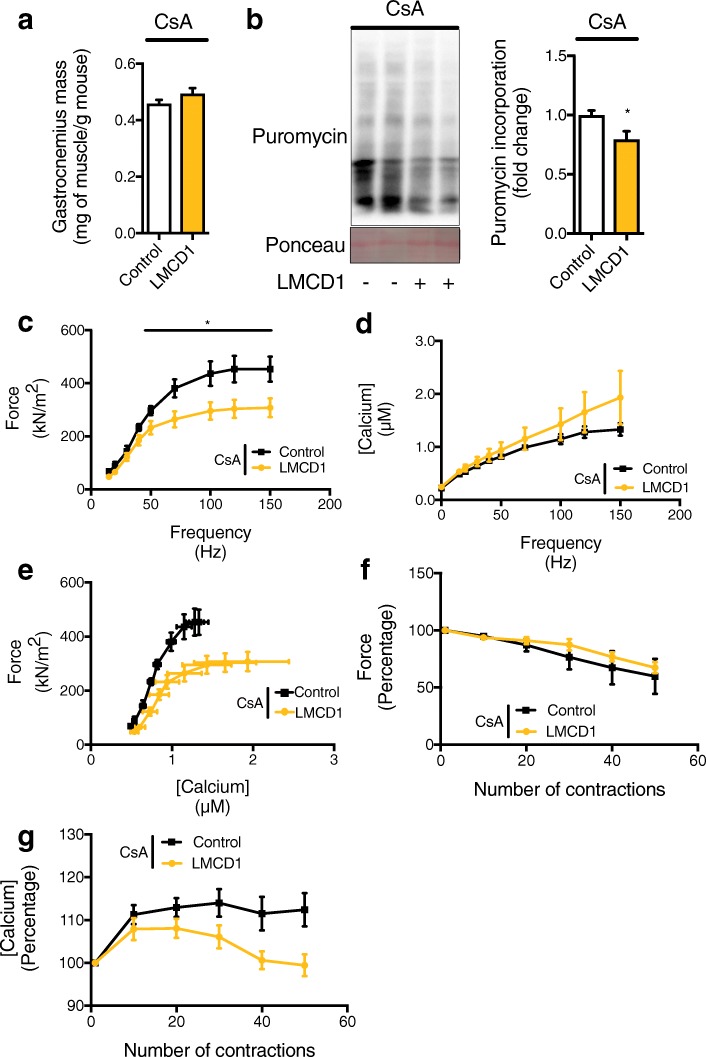
LMCD1 depends on calcineurin signaling to increase protein synthesis, force and calcium handling. **a** Gastrocnemius mass normalized by body weight after 7 days of GFP (control) or Lmcd1 expression and cyclosporine A (CsA) i.p. injection (*n* = 6). **b** In vivo muscle protein synthesis determination by puromycin incorporation in mice treated as in (**a**), and respective quantification (*n* = 6). **c** Force normalized for the cross-sectional area (specific force) for the different frequency tested (15–150 Hz) at 1-min interval in mice treated as in (**a**) (*n* = 10). **d** Myoplasmic free Ca^2+^ concentration for the different frequency tested (15–150 Hz) at 1-min interval in mice treated as in (**a**) (*n* = 10). **e** Specific force and myoplasmic free Ca^2+^ concentration for the mean of the fibers for the different frequencies tested (15–150 Hz) at 1-min intervals. Mice treated as in (**a**) (*n* = 10). **f** Percentage of force relative to the first contraction. Peak force was measured at 70 Hz tetani of 350 ms duration given at 2-s intervals for 50 contractions in mice treated as in (**a**) (*n* = 10). **g** Percentage of myoplasic free Ca^2+^ relative to the first contraction. Peak myoplasmic free Ca^2+^ concentration was measured at 70 Hz tetani of 350-ms duration given at 2-s intervals for 50 contractions in mice treated as in (**a**) (*n* = 10). Data is shown as mean ± SEM and *p < 0.05

To determine if calcineurin is downstream of LMCD1 in the induction of skeletal muscle hypertrophy, we ectopically expressed *Lmcd1* in the *gastrocnemius* muscle (or GFP in the contralateral limb, as before), and treated those mice with cyclosporine A (CsA). Strikingly, treatment with CsA completely abolished the LMCD1-mediated increase in muscle mass (Fig. 7a) and decreased protein synthesis (Fig. 7b). In addition, FDB fibers overexpressing *Lmcd1* and treated with CsA showed a higher Ca^2+^ requirement to achieve the same specific force as the control fibers (Fig. 7c–e), with a fatigue phenotype similar with the control fibers (Fig. 7f–g). Those results were similar to the ones obtained when silencing *Lmcd1* confirming the role of calcineurin in LMCD1 actions regulating skeletal muscle hypertrophy, force, and intracellular calcium handling.

### LMCD1 interacts with Calcineurin to inhibit MLN activity and improve Ca^2+^ handling

**Fig. 8:**
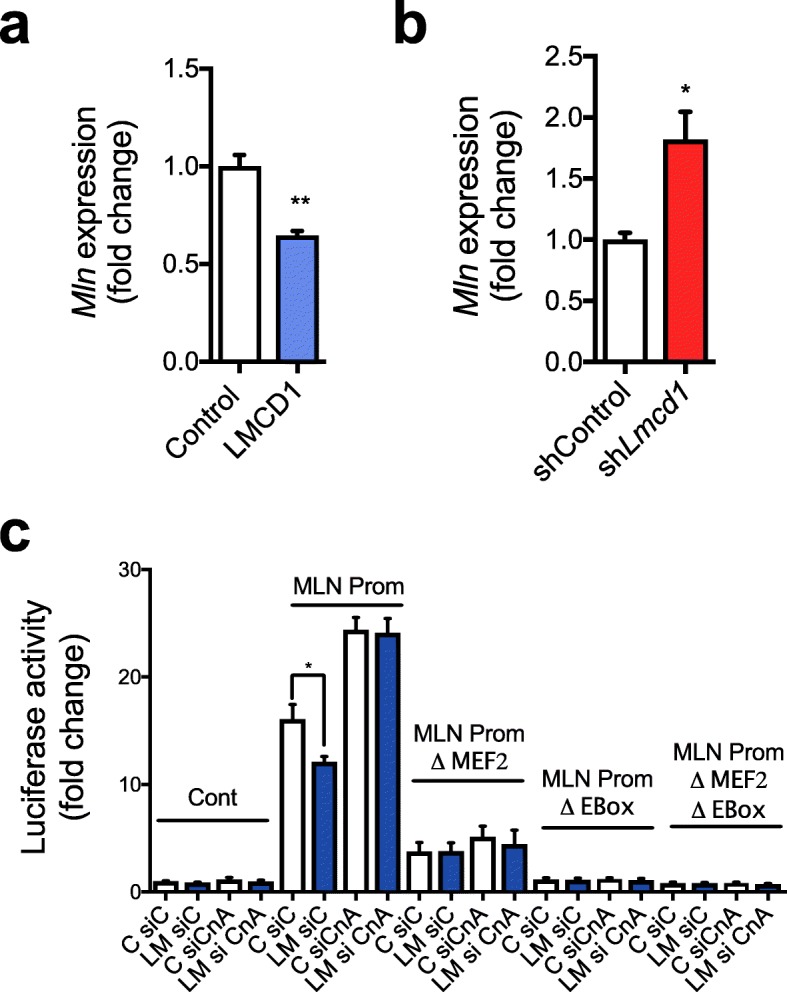
LMCD1 regulates myoregulin (Mln) expression and depends on calcineurin signaling. **a** qRT-PCR of *Mln* in gastrocnemius after 7 days of GFP (control) or LMCD1 expression (*n* = 6). **b** qRT-PCR *Mln* in gastrocnemius after 7 days of shControl or sh*Lmcd1* delivery to muscle (*n* = 6). **c** Luciferase activity of control vector or MLN promoter (with no mutations, mutations on the myocyte enhancer factor-2 (MEF2) domain, on the enhancer box (EBox) domain or in both domains) in C2C12 cells transfected with scrambled siRNA or siCalcineurin (siCnA) and 2 days after transduction with GFP (control) or LMCD1 adenovirus (*n* = 5). Data is shown as mean ± SEM and *p < 0.05; **p < 0.01

The effects of LMCD1 on Ca^2+^ handling we observed are reminiscent of what has been previously reported for *Mln*-knockout (KO) mice, which show better exercise performance and no changes in gene expression related to SERCA and ryanodine receptor 1. Since MLN has been shown to negatively regulate SERCA pump activity [10], we investigated if LMCD1 affects *Mln* expression. We observed that *Lmcd1* expression does indeed decrease *Mln* transcript levels (Fig. 8a). In addition, *Mln* expression is increased upon *Lmcd1* silencing, further highlighting the strong connection between these two proteins (Fig. 8b). Furthermore, *Lmcd1* expression was sufficient to decrease the activity of a luciferase reporter vector containing a *Mln* gene promoter fragment [10], (Fig. 8c). This effect was abrogated when calcineurin was silenced (Fig. 8c), suggesting that LMCD1 together with calcineurin regulate MLN expression and calcium handling. Together, our results indicate that LMCD1 is an important regulator of muscle mass and function. Through a series of gain- and loss-of-function studies, we show that LMCD1 controls protein synthesis, muscle fiber size, and increases specific force and Ca^2+^ handling.

## Discussion

Here, we show that LMCD1 increases skeletal muscle protein synthesis and mass, coupled to increased specific force with less Ca^2+^ requirement. Although the biological roles of LMCD1 remain fairly uncharacterized, this protein has been shown to have both cytosolic and nuclear activities. LMCD1 has been shown to induce cardiac hypertrophy [8, 9] and mutations in the LMCD1 gene have been involved in the regulation of hepatocellular carcinoma cell migration [21]. However, to date, no role for LMCD1 has been described in skeletal muscle.

Muscle hypertrophy induced by LMCD1 seems to occur mainly through increased protein synthesis. Our results show an increase in total and phosphorylated AKT and S6 levels, but a decrease in 4E-BP1 levels (Fig. 4d, Fig. 6a, and Additional file 3: Figure S3a). This separation between S6 and 4E-BP1 phosphorylation in the context of muscle hypertrophy has been previously reported [22]. Indeed, S6 kinase and 4E-BP1 seem to have compensatory roles in the regulation of protein synthesis, and they do not always act in concert to induce hypertrophy. Mice lacking 4E-BP1 show no changes in muscle growth but become completely dependent on S6 kinase to maintain protein synthesis and muscle mass [23]. On the other hand, S6 kinase-independent muscle growth can occur through 4E-BP1. However, S6 kinase KO mice have a crucial decrease in specific muscle force, and defective ribosome biogenesis [22]. In this context, and although the effects on gene expression that we observed upon *Lmcd1* expression were mild, we could determine an increase in *Pgc*-*1α4* expression. PGC-1α4 has been described to increase *Igf-1* and decrease *myostatin* expression to induce skeletal muscle hypertrophy [6]. However, this happens without affecting muscle intrinsic force, suggesting LMCD1 acts through additional mechanisms. Additionally, the effects of LMCD1 might be dependent on muscle fiber type composition, as the amplitude of hypertrophy varied between different muscles (Fig. 2c and Additional file 3: Figure S1a). Constitutively active AKT has been shown to be sufficient to induce skeletal muscle hypertrophy [24, 25]. LMCD1 gain- and loss-of-function correlated positively with the expression and phosphorylation of AKT (Fig. 4d and Fig. 6a, e), supporting the role of LMCD1 as a regulator of protein synthesis. In addition, LMCD1 increased the phosphorylation of IRS1, which has been reported as the initial IGF1 signal to activate AKT [4]. Moreover, calcineurin has been shown to regulate IRS2 in part through the nuclear factor of activated T cells (NFAT), as NFAT has been shown to occupy the *IRS2* promoter in a calcineurin-sensitive manner [26]. LMCD1 also decreased the expression of *Murf1*, which has been described to induce muscle atrophy by binding and inducing degradation of Titin. Although the effects of LMCD1 on *Murf1* expression were mild, they could potentially contribute to the increased muscle mass [27]. Finally, our results suggest that LMCD1 is sufficient but not necessary to improve muscle force, calcium handling, and fatigue-resistance. Although loss-of-function does not always mirror the results of gain-of-function, it is possible that a complete LMCD1 knockout could result in more robust effects.

Our results show that LMCD1-induced hypertrophy is strictly dependent on calcium and calcineurin. Indeed, calcineurin inhibition by CsA was enough to block skeletal muscle hypertrophy, when overexpressing LMCD1. Interestingly, in these conditions, we observed significant muscle damage (data not shown), decreased muscle size, and protein synthesis, which correlated with a decrease in force and calcium handling (Fig. 7). These results suggest that cyclosporin treatment could lead to muscle damage in situations that induce LMCD1 (e.g., combined exercise training). In line with this, there are some reports that patients under CsA treatment showed muscle impairments/damage [28, 29]. Calcineurin activation has been suggested to block the nuclear localization of FOXO transcription factors and the expression of several FOXO-targeted genes such as *Murf1* and atrogin [30]. Interestingly, FOXO signaling was also changed after LMCD1 overexpression (Fig. 4c).

However, the importance of calcineurin for muscle hypertrophy has been debated. While some authors report that calcineurin levels and/or activity do not correlate with skeletal muscle hypertrophy (in a rat model of muscle synergistic ablation) [31], others observe the opposite using quite similar systems [32]. Also, genetic deletion of calcineurin A or B does not change baseline muscle mass [33]. In contrast, overexpression of IGF1 in C2C12 cells induces myotube hypertrophy, which is abolished by CsA [34]. In addition, it was demonstrated that muscle expression of a constitutively active form of calcineurin induces muscle hypertrophy [35]. Interestingly, that mouse model (MCK-CN*), [35] shows the same fiber type switch we observe upon *Lmcd1* expression in muscle, i.e., an increase of type I, IIa, and IIx fibers at the expense of IIb (Fig. 1k).

Efficient Ca^2+^ release and reuptake from the SR is critical for muscle performance. Conversely, situations in which these processes are dysregulated induce an impairment in slowing muscle relaxation or contraction, resulting in accelerated fatigue [36]. In this context, the phenotype we observed upon manipulating *Lmcd1* levels recapitulates what has been previously for the recently described micropeptide MLN. MLN regulates intracellular Ca^2+^ levels by interacting directly with SERCA, and reducing Ca^2+^ uptake into the SR. Thus, *Mln*-KO mice exhibit improved Ca^2+^ handling in skeletal muscle and exercise performance [10]. Since LMCD1 regulates the expression of *Mln* (Fig. 8a, b), we propose that it regulates Ca^2+^ handling by reducing MLN activity. In fact, when using a luciferase reporter vector with a *Mln* promoter fragment, we could confirm that in the absence of calcineurin, LMCD1 is not capable of affecting reporter gene activity (Fig. 8c). By reducing *Mln* levels, LMCD1 de-represses SERCA function, increases Ca^2+^ influx, and leads to decreased fatigue and higher force with less Ca^2+^ requirement. These changes in Ca^2+^ can also activate even more calcineurin and improve protein synthesis. In agreement, when we silenced *Lmcd1*, we observed increased *Mln* expression, and lower protein synthesis, force, and Ca^2+^ handling.

## Conclusions

Here, we show that LMCD1 is reduced in skeletal muscle of patients with diseases such as Duchenne muscular dystrophy and myotonic dystrophy. According to our results, decreasing LMCD1 leads to increased *Mln* expression and decreased Ca^2+^ handling, force, and skeletal muscle size. This suggests that the reduction in *Lmcd1* expression may play an important role in skeletal muscle disease. On the other hand, *Lmcd1* expression increases protein synthesis and muscle fiber size in vivo and in vitro. Notably, LMCD1 proved to be sufficient to increase specific force with lower requirement for Ca^2+^ handling. This work uncovers a novel role for LMCD1 in the govern of skeletal muscle size, force, and Ca^2+^ handling with potential therapeutic implications.

## Supplementary information

**Additional file 1: Table S1.** Primers used for detection of gene expression by qRT-PCR.

**Additional file 2: Table S2.** Kinase activities after LMCD1 overexpression in differentiated mouse primary myotubes.

**Additional file 3: Figure S1.** LMCD1 induces small changes in gene expression of genes related with sarcoplasmic reticulum (SR) stress and muscle structural proteins. **Figure S2**. Silencing *Lmcd1* does not change gene expression. **Figure S3**. LMCD1 has mainly cytosolic location and induces small changes in gene expression in differentiated primary mouse myotubes.

## Data Availability

*Lmcd1* mRNA expression in different available datasets of human diseases was compared with healthy subjects (Accession Numbers GSE3307, GSE7014, E-MEXP-2681, GSE10760, GSE1007, GSE11971, GSE39454).

## Abbreviations

CsA: Cyclosporine A
FDB: Flexor digitorum brevis
GFP: Green fluorescent protein
IGF-1: Insulin-like growth factor 1
IRS1: Insulin receptor substrate 1
LMCD1: LIM and cysteine-rich domains 1
MLN: Myoregulin
mTOR: Mammalian target of rapamycin
MURF1: Muscle RING-finger protein-1
MyHC: Myosin heavy chain
PGC-1α: Peroxisome proliferator-activated receptor gamma coactivator-1alpha
SERCA: Sarcoplasmic/endoplasmic reticulum calcium ATPase
SR: Sarcoplasmic reticulum

## References

[1] Frontera WR, Ochala J (2015). Skeletal muscle: a brief review of structure and function. Calcif Tissue Int.

[2] Qaisar R, Renaud G, Morine K, Barton ER, Sweeney HL, Larsson L (2012). Is functional hypertrophy and specific force coupled with the addition of myonuclei at the single muscle fiber level?. FASEB J.

[3] Amthor H, Macharia R, Navarrete R, Schuelke M, Brown SC, Otto A, Voit T, Muntoni F, Vrbova G, Partridge T, Zammit P, Bunger L, Patel K (2007). Lack of myostatin results in excessive muscle growth but impaired force generation. Proc Natl Acad Sci U S A.

[4] Schiaffino S, Dyar KA, Ciciliot S, Blaauw B, Sandri M (2013). Mechanisms regulating skeletal muscle growth and atrophy. FEBS J.

[5] Lee SJ (2004). Regulation of muscle mass by myostatin. Annu Rev Cell Dev Biol.

[6] Ruas JL, White JP, Rao RR, Kleiner S, Brannan KT, Harrison BC, Greene NP, Wu J, Estall JL, Irving BA, Lanza IR, Rasbach KA, Okutsu M, Nair KS, Yan Z, Leinwand LA, Spiegelman BM (2012). A PGC-1alpha isoform induced by resistance training regulates skeletal muscle hypertrophy. Cell.

[7] Rath N, Wang Z, Lu MM, Morrisey EE (2005). LMCD1/Dyxin is a novel transcriptional cofactor that restricts GATA6 function by inhibiting DNA binding. Mol Cell Biol.

[8] Frank D, Frauen R, Hanselmann C, Kuhn C, Will R, Gantenberg J, Fuzesi L, Katus HA, Frey N (2010). Lmcd1/Dyxin, a novel Z-disc associated LIM protein, mediates cardiac hypertrophy in vitro and in vivo. J Mol Cell Cardiol.

[9] Bian ZY, Huang H, Jiang H, Shen DF, Yan L, Zhu LH, Wang L, Cao F, Liu C, Tang QZ, Li H (2010). LIM and cysteine-rich domains 1 regulates cardiac hypertrophy by targeting calcineurin/nuclear factor of activated T cells signaling. Hypertension.

[10] Anderson DM, Anderson KM, Chang CL, Makarewich CA, Nelson BR, McAnally JR, Kasaragod P, Shelton JM, Liou J, Bassel-Duby R, Olson EN (2015). A micropeptide encoded by a putative long noncoding RNA regulates muscle performance. Cell.

[11] Sczelecki S, Besse-Patin A, Abboud A, Kleiner S, Laznik-Bogoslavski D, Wrann CD, Ruas JL, Haibe-Kains B, Estall JL (2014). Loss of Pgc-1alpha expression in aging mouse muscle potentiates glucose intolerance and systemic inflammation. Am J Physiol Endocrinol Metab.

[12] Robinson MM, Dasari S, Konopka AR, Johnson ML, Manjunatha S, Esponda RR, Carter RE, Lanza IR, Nair KS (2017). Enhanced protein translation underlies improved metabolic and physical adaptations to different exercise training modes in young and old humans. Cell Metab.

[13] Martinez-Redondo V, Jannig PR, Correia JC, Ferreira DM, Cervenka I, Lindvall JM, Sinha I, Izadi M, Pettersson-Klein AT, Agudelo LZ, Gimenez-Cassina A, Brum PC, Dahlman-Wright K, Ruas JL (2016). Peroxisome proliferator-activated receptor gamma coactivator-1 alpha isoforms selectively regulate multiple splicing events on target genes. J Biol Chem.

[14] Cheng AJ, Westerblad H (2017). Mechanical isolation, and measurement of force and myoplasmic free [Ca (2+)] in fully intact single skeletal muscle fibers. Nat Protoc.

[15] Andrade FH, Reid MB, Allen DG, Westerblad H (1998). Effect of hydrogen peroxide and dithiothreitol on contractile function of single skeletal muscle fibres from the mouse. J Physiol.

[16] Bespalova IN, Burmeister M (2000). Identification of a novel LIM domain gene, LMCD1, and chromosomal localization in human and mouse. Genomics.

[17] Blackwell Thomas A., Cervenka Igor, Khatri Bhuwan, Brown Jacob L., Rosa-Caldwell Megan E., Lee David E., Perry Richard A., Brown Lemuel A., Haynie Wesley S., Wiggs Michael P., Bottje Walter G., Washington Tyrone A., Kong Byungwhi C., Ruas Jorge L., Greene Nicholas P. (2018). Transcriptomic analysis of the development of skeletal muscle atrophy in cancer-cachexia in tumor-bearing mice. Physiological Genomics.

[18] Cargnello M, Roux PP (2011). Activation and function of the MAPKs and their substrates, the MAPK-activated protein kinases. Microbiol Mol Biol Rev.

[19] Yoon MS (2017). mTOR as a key regulator in maintaining skeletal muscle mass. Front Physiol.

[20] Bruno NE, Kelly KA, Hawkins R, Bramah-Lawani M, Amelio AL, Nwachukwu JC, Nettles KW, Conkright MD (2014). Creb coactivators direct anabolic responses and enhance performance of skeletal muscle. EMBO J.

[21] Chang CY, Lin SC, Su WH, Ho CM, Jou YS (2012). Somatic LMCD1 mutations promoted cell migration and tumor metastasis in hepatocellular carcinoma. Oncogene.

[22] Marabita M, Baraldo M, Solagna F, Ceelen JJM, Sartori R, Nolte H, Nemazanyy I, Pyronnet S, Kruger M, Pende M, Blaauw B (2016). S6K1 is required for increasing skeletal muscle force during hypertrophy. Cell Rep.

[23] Le Bacquer O, Petroulakis E, Paglialunga S, Poulin F, Richard D, Cianflone K, Sonenberg N (2007). Elevated sensitivity to diet-induced obesity and insulin resistance in mice lacking 4E-BP1 and 4E-BP2. J Clin Invest.

[24] Pallafacchina G, Calabria E, Serrano AL, Kalhovde JM, Schiaffino S (2002). A protein kinase B-dependent and rapamycin-sensitive pathway controls skeletal muscle growth but not fiber type specification. Proc Natl Acad Sci U S A.

[25] Blaauw B, Canato M, Agatea L, Toniolo L, Mammucari C, Masiero E, Abraham R, Sandri M, Schiaffino S, Reggiani C (2009). Inducible activation of Akt increases skeletal muscle mass and force without satellite cell activation. FASEB J.

[26] Soleimanpour SA, Crutchlow MF, Ferrari AM, Raum JC, Groff DN, Rankin MM, Liu C, De Leon DD, Naji A, Kushner JA, Stoffers DA (2010). Calcineurin signaling regulates human islet {beta}-cell survival. J Biol Chem.

[27] Centner T, Yano J, Kimura E, McElhinny AS, Pelin K, Witt CC, Bang ML, Trombitas K, Granzier H, Gregorio CC, Sorimachi H, Labeit S (2001). Identification of muscle specific ring finger proteins as potential regulators of the titin kinase domain. J Mol Biol.

[28] Ding H, Li Z, Zhang J (2019). A case report of cyclosporine-induced myopathy with subacute muscular atrophy as initial presentation. Medicine (Baltimore).

[29] Iaccarino L, Bartoloni E, Gerli R, Alunno A, Barsotti S, Cafaro G, Gatto M, Talarico R, Tripoli A, Zen M, Neri R, Doria A (2014). Drugs in induction and treatment of idiopathic inflammatory myopathies. Auto Immun Highlights.

[30] Lara-Pezzi E, Winn N, Paul A, McCullagh K, Slominsky E, Santini MP, Mourkioti F, Sarathchandra P, Fukushima S, Suzuki K, Rosenthal N (2007). A naturally occurring calcineurin variant inhibits FoxO activity and enhances skeletal muscle regeneration. J Cell Biol.

[31] Bodine SC, Stitt TN, Gonzalez M, Kline WO, Stover GL, Bauerlein R, Zlotchenko E, Scrimgeour A, Lawrence JC, Glass DJ, Yancopoulos GD (2001). Akt/mTOR pathway is a crucial regulator of skeletal muscle hypertrophy and can prevent muscle atrophy in vivo. Nat Cell Biol.

[32] Dunn SE, Burns JL, Michel RN (1999). Calcineurin is required for skeletal muscle hypertrophy. J Biol Chem.

[33] Parsons SA, Wilkins BJ, Molkentin JD, Bueno OF (2003). Altered skeletal muscle phenotypes in calcineurin Aalpha and Abeta gene-targeted mice. Mol Cell Biol.

[34] Semsarian C, Wu MJ, Ju YK, Marciniec T, Yeoh T, Allen DG, Harvey RP, Graham RM (1999). Skeletal muscle hypertrophy is mediated by a Ca2+-dependent calcineurin signalling pathway. Nature.

[35] Talmadge RJ, Otis JS, Rittler MR, Garcia ND, Spencer SR, Lees SJ, Naya FJ (2004). Calcineurin activation influences muscle phenotype in a muscle-specific fashion. BMC Cell Biol.

[36] Allen DG, Lamb GD, Westerblad H (2008). Impaired calcium release during fatigue. J Appl Physiol (1985).

