# Supplementary material for: LIM and cysteine-rich domains 1 (LMCD1) regulates skeletal muscle hypertrophy, calcium handling, and force
Source: Skelet Muscle. 2019 Oct 31;9:26. doi: 10.1186/s13395-019-0214-1 (PMC6822430; doi:10.1186/s13395-019-0214-1)
Supplement: Supplementary file 3 — Additional file 3: Figure S1. LMCD1 induces small changes in gene expression of genes related with sarcoplasmic reticulum (SR) stress and muscle structural proteins. Figure S2. Silencing Lmcd1 does not change gene expression. Figure S3. LMCD1 has mainly cytosolic location and induces small changes in gene expression in differentiated primary mouse myotubes. [file 13395_2019_214_MOESM3_ESM.pdf]

Supplementary Figures for

LIM and Cysteine-rich Domains 1 (LMCD1) regulates skeletal muscle hypertrophy, calcium handling, and force

Duarte M.S. Ferreira[1], Arthur J. Cheng[2], Leandro Z. Agudelo[1], Igor Cervenka[1], Thomas Chaillou[2], Jorge C. Correia[1], Margareta Porsmyr-Palmertz[1], Manizheh Izadi[1], Alicia Hansson[1], Vicente Martínez-Redondo[1], Paula Valente-Silva[1], Amanda T. Petersson-Klein[1], Jennifer L. Estall[3,4,5], Matthew M. Robinson[6], K. Sreekumaran Nair[6], Johanna T. Lanner[2], and Jorge L. Ruas[1]

Corresponding author:

Jorge L. Ruas

Email: Jorge.Ruas@ki.se

This PDF file includes:

Figure S1
Figure S2
Figure S3
Table S1

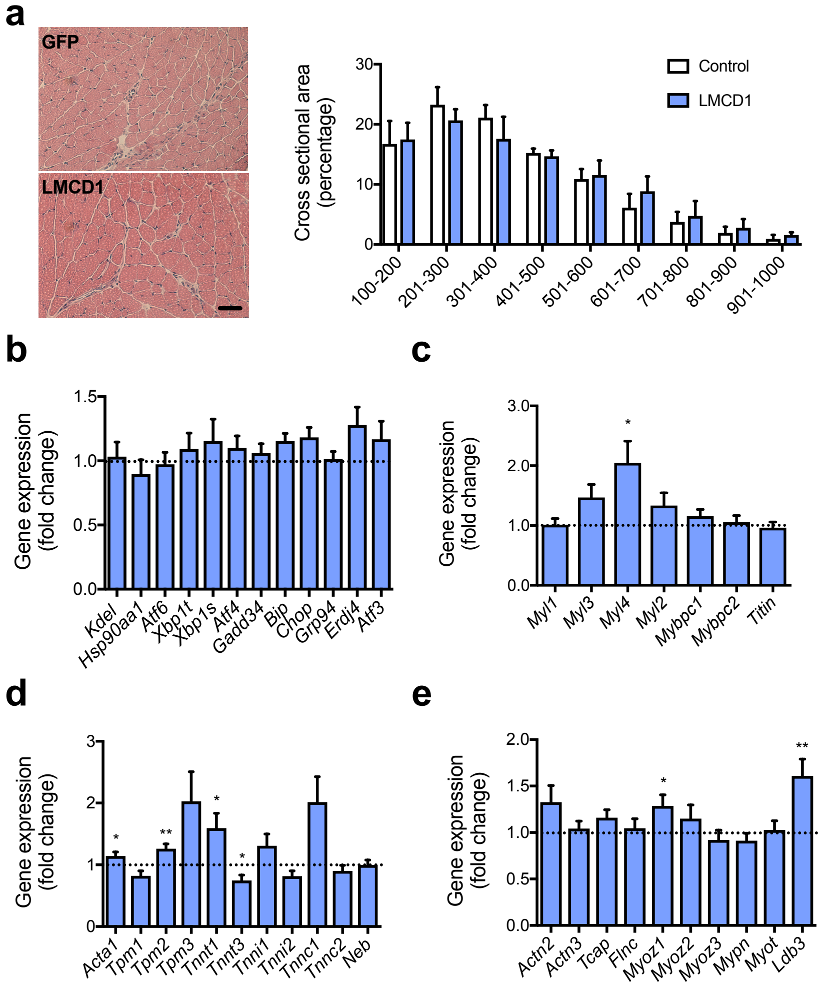

Figure S1. LMCD1 induces small changes in gene expression of genes related with sarcoplasmic reticulum (SR) stress and muscle structural proteins. **(a)** Determination of fiber cross sectional area. Hematoxylin and eosin staining of sections from *gastrocnemius* muscle 7 days after GFP (control) or LMCD1 adenovirus injection and respective percentage of fiber sectional area (n = 3). **(b)** Quantitative RT-PCR (qRT-PCR) of genes involved in SR stress in in mice treated as in (*a*) (n = 6). **(c)** qRT-PCR of genes involved in the expression of proteins from the thick filaments in *gastrocnemius* muscle in mice treated as in (*a*) (n = 6). **(d)** qRT-PCR of genes involved in the expression of proteins from the thin filaments in *gastrocnemius* muscle in mice treated as in (*a*) (n = 6). **(e)** qRT-PCR of genes involved in the expression of proteins present in the Z-disk in *gastrocnemius* muscle in mice treated as in (*a*) (n = 6). Data is shown as mean ± SEM and * $p < 0.05$; ** $p < 0.01$. Abbreviations: *Kdel*, (Lys-Asp-Glu-Leu) Endoplasmic Reticulum Protein Retention Receptor 1; *Hsp90aa1*, Heat Shock Protein 90 Alpha Family Class A Member 1; *Atf6*, Activating Transcription Factor 6; *Xbp1t*, X-box binding protein 1 total; *Xbp1s*, X-box binding protein 1 short; *Atf4*, Activating Transcription Factor 4; *Gadd34*, Growth arrest and DNA damage-inducible protein 34; *Bip*, Binding immunoglobulin protein; *Chop*, C/EBP homologous protein; *Grp94*, 94 kDa

glucose- regulated protein; *Erdj4*, Endoplasmic Reticulum DNA J Domain-Containing Protein 4; *Atf3*, Activating Transcription Factor 3; *Myl1*, Myosin light chain 1; *Myl3*, Myosin light chain 3; *Myl4*, Myosin light chain 4; *Myl2*, Myosin light chain 2; *Mybpc1*, Myosin Binding Protein C 1; *Mybpc2*, Myosin Binding Protein C 2; *Acta1*, Actin, Alpha 1; *Tpm1*, Tropomyosin 1; *Tpm2*, Tropomyosin 2; *Tpm3*, Tropomyosin 3; *Tnnt1*; Troponin T1; *Tnnt3*, Troponin T3; *Tnni1*, Troponin I1; *Tnni2*, Troponin I2; *Tnnc1*, Troponin C1; *Tnnc2*, Troponin C2; *Neb*, Nebulin; *Actn2*, Actinin Alpha 2; *Actn3*, Actinin Alpha 2; *Tcap*, Telethonin; *Flnc*, Filamin C; *Myoz1*, Myozenin 1; *Myoz2*, Myozenin 2; *Myoz3*, Myozenin 3; *Mypn*, Myopalladin; *Myot*, Myotilin; *Ldb3*, LIM Domain Binding 3.

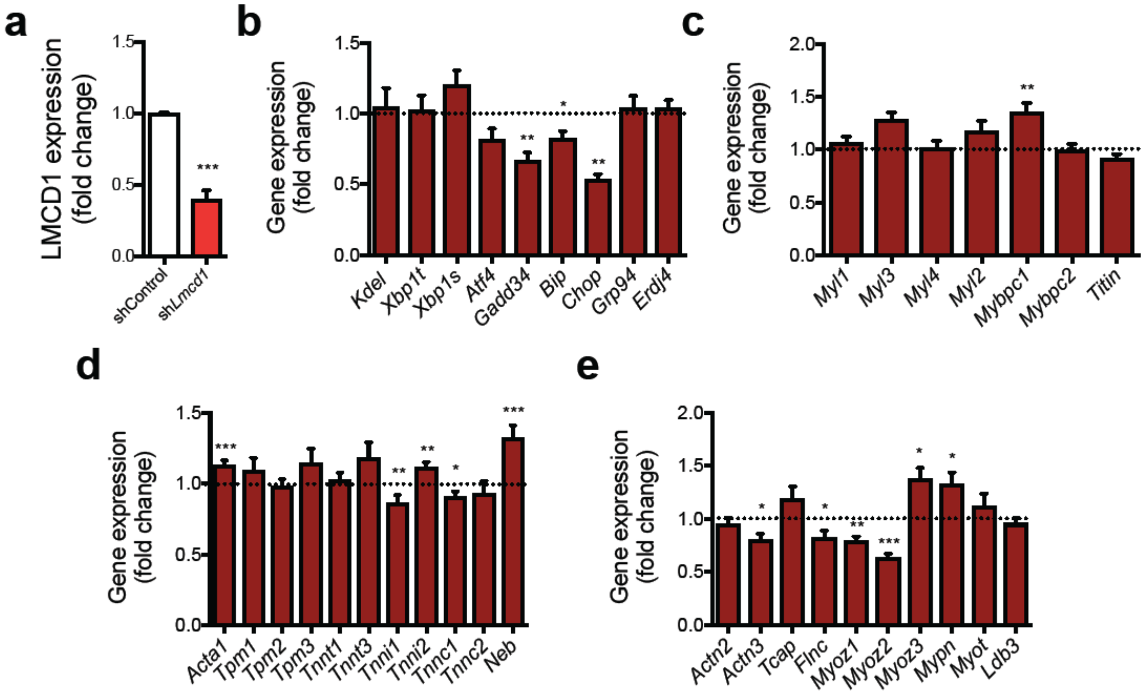

Figure S2. Silencing *Lmcd1* does not change gene expression. **(a)** qRT-PCR for *Lmcd1* in *gastrocnemius* 7 days after intramuscular delivery of a scrambled/control shRNA or a sh*Lmcd1* (n = 6). **(b)** qRT-PCR of genes involved in SR stress in differentiated primary mouse myotubes after 2 days transduction of scrambled shRNA or sh*Lmcd1* adenovirus (*n* = 8). **(c)** qRT-PCR of genes involved in the expression of proteins from the thick filaments in differentiated primary mouse myotubes transduced as in (a) (*n* = 8). **(d)** qRT-PCR of genes involved in the expression of proteins from the thin filaments in differentiated primary mouse myotubes transduced as in (a) (*n* = 8). **(e)** qRT-PCR of genes involved in the expression of proteins present in the Z-disk in differentiated primary mouse myotubes transduced as in (a) (*n* = 8). Data is shown as mean ± SD and * $p < 0.05$; ** $p < 0.01$; *** $p < 0.001$. Abbreviations: *Kdel*, (Lys-Asp-Glu-Leu) Endoplasmic Reticulum Protein Retention Receptor 1; *Hsp90aa1*, Heat Shock Protein 90 Alpha Family Class A Member 1; *Atf6*, Activating Transcription Factor 6; *Xbp1t*, X-box binding protein 1 total; *Xbp1s*, X-box binding protein 1 short; *Atf4*,

Activating Transcription Factor 4; *Gadd34*, Growth arrest and DNA damage-inducible protein 34; *Bip*, Binding immunoglobulin protein; *Chop*, C/EBP homologous protein; *Grp94*, 94 kDa glucose- regulated protein; *Erdj4*, Endoplasmic Reticulum DNA J Domain-Containing Protein 4; *Myl1*, Myosin light chain 1; *Myl3*, Myosin light chain 3; *Myl4*, Myosin light chain 4; *Myl2*, Myosin light chain 2; *Mybpc1*, Myosin Binding Protein C 1; *Mybpc2*, Myosin Binding Protein C 2; *Acta1*, Actin, Alpha 1; *Tpm1*, Tropomyosin 1; *Tpm2*, Tropomyosin 2; *Tpm3*, Tropomyosin 3; *Tnnt1*; Troponin T1; *Tnnt3*, Troponin T3; *Tnni1*, Troponin I1; *Tnni2*, Troponin I2; *Tnnc1*, Troponin C1; *Tnnc2*, Troponin C2; *Neb*, Nebulin; *Actn2*, Actinin Alpha 2; *Actn3*, Actinin Alpha 2; *Tcap*, Telethonin; *Flnc*, Filamin C; *Myoz1*, Myozenin 1; *Myoz2*, Myozenin 2; *Myoz3*, Myozenin 3; *Mypn*, Myopalladin; *Myot*, Myotilin; *Ldb3*, LIM Domain Binding 3.

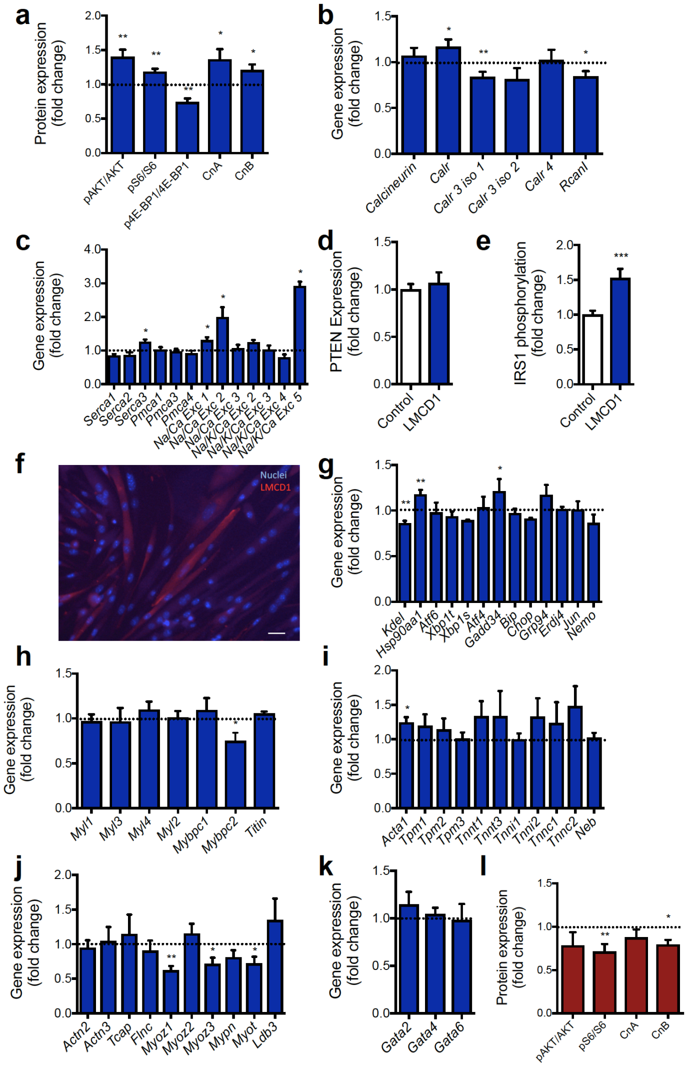

Figure S3. LMCD1 has mainly cytosolic location and induces small changes in gene expression in differentiated primary mouse myotubes. **(a)** Western blot

quantification for AKT Ser^{473} phosphorylation, S6 Ser^{235} phosphorylation, 4E-BP1 Thr^{37}phosphorylation, calcineurin A and calcineurin B in differentiated primary mouse myotubes after 2 days of transduction with GFP (dotted line) or LMCD1 adenovirus (*n* = 8). **(b)** qRT-PCR of genes involved in the expression of Ca^{2+} related proteins in differentiated primary mouse myotubes treated as in (a) (*n* = 8). **(c)** qRT-PCR of genes involved in the expression of Ca^{2+} channels proteins in differentiated primary mouse myotubes and transduced as in (a) (*n* = 8). **(d)** Western blot quantification for PTEN in differentiated myotubes transduced as in (a) (*n* = 6). **(e)** Western blot quantification for IRS1 Tyr^{612} phosphorylation in differentiated myotubes transduced as in (a) (*n* = 4). **(f)** Representative microscopy image of immunocytochemistry against LMCD1 in differentiated primary mouse myotubes. DAPI staining was used to stain nuclei (200 x; *n* = 6). Scale bar = 50 µm. **(g)** qRT-PCR of genes involved in SR stress in differentiated primary mouse myotubes transduced as in (a) (*n* = 8). **(h)** qRT-PCR of genes involved in the expression of proteins from the thick filaments in differentiated primary mouse myotubes transduced as in (a) (*n* = 8). **(i)** qRT-PCR of genes involved in the expression of proteins from the thin filaments in differentiated primary mouse myotubes transduced as in (a) (*n* = 8). **(j)** qRT-PCR of genes involved in the expression of proteins present in the Z-disk in differentiated primary mouse myotubes transduced as in (a) (*n* = 8). **(k)** qRT-PCR of *Gata* genes in differentiated primary mouse myotubes transduced as in (a) (*n* = 8). **(l)** Western blot quantification for AKT Ser^{473} phosphorylation, S6 Ser^{235} phosphorylation, calcineurin A and calcineurin B in differentiated primary mouse myotubes after 2 days transduction of scrambled shRNA or sh*Lmcd1* adenovirus (*n* = 8). Data is shown as mean ± SD and * $p < 0.05$; ** $p < 0.01$; *** $p < 0.001$. Abbreviations: AKT, Protein kinase B; 4E-BP1, Eukaryotic translation initiation factor 4E-binding protein 1; CnA, Calcineurin A; CnB, Calcineurin B; *Calr*, Calreticulin; *RcanI*, Calcipressin 1; *Serca*, Sarco/Endoplasmic Reticulum Ca^{2+}-ATPase; *Pmca*, Plasma Membrane Ca^{2+}-ATPase; *Kdel*, (Lys-Asp-Glu-Leu) Endoplasmic Reticulum Protein Retention Receptor 1; *Hsp90aa1*, Heat Shock Protein 90 Alpha Family Class A Member 1; *Atf6*, Activating Transcription Factor 6; *Xbp1t*, X-box binding protein 1 total; *Xbp1s*, X-box binding protein 1 short; *Atf4*, Activating Transcription Factor 4; *Gadd34*, Growth arrest and DNA damage-inducible protein 34; *Bip*, Binding immunoglobulin protein; *Chop*, C/EBP homologous protein; *Grp94*, 94 kDa glucose- regulated protein; *Erdj4*, Endoplasmic Reticulum DNA J Domain-Containing Protein 4; *Nemo*, NF-κB essential modulator; *Myl1*, Myosin light chain 1; *Myl3*, Myosin light chain 3; *Myl4*, Myosin light chain 4; *Myl2*, Myosin light chain 2; *Mybpc1*, Myosin Binding Protein C 1; *Mybpc2*, Myosin Binding Protein C 2; *Acta1*, Actin, Alpha 1; *Tpm1*, Tropomyosin 1; *Tpm2*, Tropomyosin 2; *Tpm3*, Tropomyosin 3; *Tnnt1*; Troponin T1; *Tnnt3*, Troponin T3; *Tnni1*, Troponin I1; *Tnni2*, Troponin I2; *Tnnc1*, Troponin C1; *Tnnc2*, Troponin C2; *Neb*, Nebulin; *Actn2*, Actinin Alpha 2; *Actn3*, Actinin Alpha 2; *Tcap*, Telethonin; *Flnc*, Filamin C; *Myoz1*, Myozenin 1; *Myoz2*, Myozenin 2; *Myoz3*, Myozenin 3; *Mypn*, Myopalladin; *Myot*, Myotilin; *Ldb3*, LIM Domain Binding 3.
